# Movement Disorders and Smart Wrist Devices: A Comprehensive Study

**DOI:** 10.3390/s25010266

**Published:** 2025-01-05

**Authors:** Andrea Caroppo, Andrea Manni, Gabriele Rescio, Anna Maria Carluccio, Pietro Aleardo Siciliano, Alessandro Leone

**Affiliations:** National Research Council of Italy, Institute for Microelectronics and Microsystems, 73100 Lecce, Italy; gabriele.rescio@cnr.it (G.R.); annamaria.carluccio@imm.cnr.it (A.M.C.); pietroaleardo.siciliano@cnr.it (P.A.S.); alessandro.leone@cnr.it (A.L.)

**Keywords:** review, smartwatch, wristband, bracelet, wrist-worn, movement disorders, Parkinson’s disease, tremors, Huntington’s disease, gait disorders, essential tremor, Tourette Syndrome, epilepsy, ataxia, seizure detection, unilateral cerebral palsy

## Abstract

In the medical field, there are several very different movement disorders, such as tremors, Parkinson’s disease, or Huntington’s disease. A wide range of motor and non-motor symptoms characterizes them. It is evident that in the modern era, the use of smart wrist devices, such as smartwatches, wristbands, and smart bracelets is spreading among all categories of people. This diffusion is justified by the limited costs, ease of use, and less invasiveness (and consequently greater acceptability) than other types of sensors used for health status monitoring. This systematic review aims to synthesize research studies using smart wrist devices for a specific class of movement disorders. Following PRISMA-S guidelines, 130 studies were selected and analyzed. For each selected study, information is provided relating to the smartwatch/wristband/bracelet model used (whether it is commercial or not), the number of end-users involved in the experimentation stage, and finally the characteristics of the benchmark dataset possibly used for testing. Moreover, some articles also reported the type of raw data extracted from the smart wrist device, the implemented designed algorithmic pipeline, and the data classification methodology. It turned out that most of the studies have been published in the last ten years, showing a growing interest in the scientific community. The selected articles mainly investigate the relationship between smart wrist devices and Parkinson’s disease. Epilepsy and seizure detection are also research topics of interest, while there are few papers analyzing gait disorders, Huntington’s Disease, ataxia, or Tourette Syndrome. However, the results of this review highlight the difficulties still present in the use of the smartwatch/wristband/bracelet for the identified categories of movement disorders, despite the advantages these technologies could bring in the dissemination of low-cost solutions usable directly within living environments and without the need for caregivers or medical personnel.

## 1. Introduction

In recent years, the number of admissions to hospitals or private nursing homes of patients suffering from various diseases has risen. Although several studies report a remarkable intensification in treatment techniques, both pharmacologically and therapeutically, this increase in patients is mainly due to the aging of the world population [1]. This motivation has progressively driven the production by technology companies of low-cost commercial wearable devices that are easy to use even for the elderly population, and that integrate sensors for acquiring data useful for vital signs estimation, activity classification, sleep quality estimation, exercise quality assessment, critical event detection, and disorders. In addition to companies, researchers are also using wearable devices to conduct scientific studies on specific clinical diseases. Such studies are favored in the modern era by the widespread and easy use of devices such as smartwatches, wristbands, or bracelets, through which it is possible to carry out large-scale data collection and analysis that would have been impossible in the past. This is a growing trend that could greatly expand our knowledge of a range of diseases.

Among the most common disorders in the world’s population are those of a neurological nature [2]. Movement disorders represent a class of neurological illnesses, responsible for aberrant movements. They could lead to slower movement execution, increased movement variability, and a decrease in functionality [3,4]. It is clear how an accurate assessment is essential for tracking the disease course in both early- and late-onset movement disorders, particularly in progressive movement disorders, as well as for evaluating and optimizing the effectiveness of treatment approaches. Currently, clinical evaluation tools including functional scales and movement disorder severity scales [5,6,7] are typically used to evaluate the impact of an intervention program or the presence and/or severity of movement disorders.

The COVID-19 pandemic that hit the population in 2020 created significant disruptions in clinical practice, the main effect of which was the spread of remote medicine to provide clinical care [8]. A consequence of this has been the advancement in scientific research for the design and engineering of technological solutions capable of providing remote clinical information through alternative methods and devices for evaluation and treatment outside the clinical environment. The rapid spread of smart wearable devices among the population has strongly contributed to the development of even remote solutions capable of providing objective and frequent information on activity recognition, detection of dangerous situations (i.e., falls), and continuous monitoring of vital signs [9,10,11].

Although the advantages of employing such devices are undoubted, their use often presents difficulties in daily use. For example, neuromuscular diagnoses and human movement studies use surface electromyography (sEMG) wearable sensors. The fields of clinical neurophysiology and electrodiagnostic medicine are where it is most utilized. The signal acquired through sEMG makes it possible to assess muscle activation, thus providing useful information for various applications such as gait and posture analysis, neurological rehabilitation, physical therapy, movement disorder analysis, and evaluation of sporting activities. However, the majority of sEMG clinical research investigations have been conducted in small groups of 10–50 participants in research institutions by engineers and biologists. Consequently, larger clinical trials are unusual because they must be conducted in medical facilities, where engineers are rarely on-site and clinical operators might not have the time, resources, or expertise to complete them [12].

Another category of wearable sensors widely investigated in the clinical area of movement disorder analysis is related to brain monitoring, and an extensively used non-invasive technique for the study of brain signals is the electroencephalogram (EEG). It works by applying conductive electrodes to the scalp to monitor the tiny electrical potentials that are produced by brain activity and emerge outside of the head. Because EEG can detect abnormal brain activity patterns, it is useful in the diagnosis of conditions such as epilepsy, sleep problems, brain tumors, and brain traumas. Also, to better understand brain function, cognitive processes, and neurological illnesses, EEG is widely used in neuroscience research, providing important insights into how the brain functions under different conditions of awareness, behavior, and tasks. EEG wearable systems generally are wired or wireless and send data to a computer by Bluetooth, wireless, or cable connection, respectively. Although wireless connections allow for greater mobility, wired EEG connections are less mobile and frequently transfer more data in each amount of time. One of the disadvantages of wireless EEG smart solutions is wireless connection stability during brain data gathering, which would result in an incomplete recording. Also, the movement of cables and electrodes can introduce distortions into the EEG signal, regardless of the kind of connection, as it can break the connections between the electrodes and the scalp. Finally, this category of devices has a low degree of acceptability, especially among elderly people, which limits their wide use [13].

With the aid of digital electronics and computer algorithms, e-textiles (also named smart or intelligent garments) are becoming more widely acknowledged for their use in the monitoring, diagnosis, and treatment of many health disorders such as movement disorders, as well as everyday activities. The various attributes of intelligent clothing vary based on the technology platform or embedded electronic components. The incorporation of electronic components gives conventional clothing new functionality that improves its capabilities and adds value for different applications [14]. Despite recent progress in the development of smart garments, their functionality, dimensions, and shapes are limited by current manufacturing processes, limiting the deployment of solutions based on the use of such technology.

Wearable smart devices such as sEMG, EEG, or smart garments belong to the category of non-commercial smart devices and are generally used for scientific research purposes. Moreover, a disadvantage of these is that their use often requires the presence of qualified personnel. Commercial wearable devices, on the other hand, are usually designed and developed to be sold to the public and worn almost continuously. To this end, they usually use sensors embedded in everyday objects, such as smartphones, smartwatches, and smart rings. These devices are relatively inexpensive, widely available in the market, and often already available to the end-user. In addition, they require no specific knowledge to be used. The drawbacks of commercial devices include their lack of advanced software and analysis algorithms, their overall lack of validation, their intended use neither for research nor clinical practice, and, with few exceptions, their inability to obtain a medical license from regulatory bodies.

A large-scale assessment of movement disorders (most of the world’s population has one) is now possible through smartphones as they integrate sensors useful for assessing disorders such as the gyroscope, accelerometer, and magnetometer as standard.

Moreover, more and more smartphones, even low-cost ones, are equipped with processing units allowing developers to design algorithmic pipelines that run directly on the smartphone in real-time. Consequently, a market has developed in recent years for applications (in some cases free of charge) that provide information on specific movement disorders. An interesting and very recent review article [15] collects and describes the apps developed for diagnosing, monitoring, assessing, or treating movement disorders.

However, the smartphone is wrongly considered a wearable device. Although for most of the day, it is held in the hand of the end-user, it is often placed within the living environment in different locations (tables, desks, bedside tables), and this is more frequent when considering the use of such devices by frail and elderly individuals. Consequently, it may be inconvenient to use smartphones to assess, for example, changes in movement disorders for which continuous monitoring is required.

Unlike the smartphone, a smart wrist device is like a wearable computer that comes in a variety of forms, dimensions, and features. Depending on the possession of these characteristics, a device is called a smartwatch, bracelet, or wristband. It is possible to comprehend the development of smartwatches in the consumer market if we take a retrospective look. The previous 30 years have seen significant growth in the electronics industry, which has altered how new technologies are applied. Furthermore, it primarily impacts small-scale equipment. One such device is the smartwatch, and its greatest feature is that it may be used to track one’s health. Most smartwatches were released nearly a decade ago, starting in 2012. Some watches were released before 2012 without an advanced OS platform.

**Figure 1:**
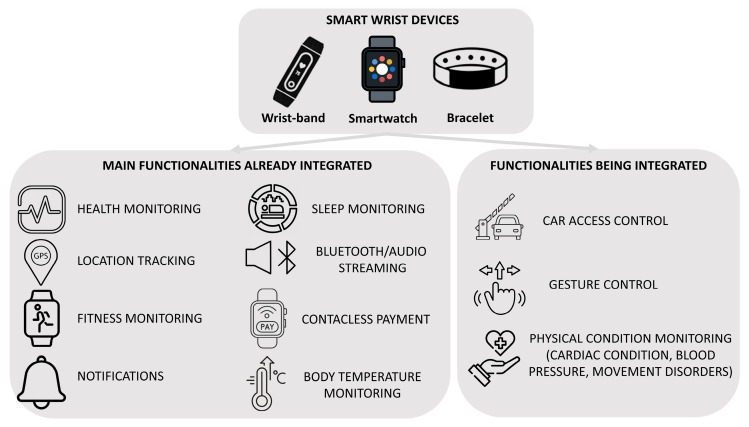
Main functionalities (already or being integrated) for smart wrist devices in the market.

A smart wrist device and a smartphone are different in several important ways. First, a smartphone is a portable device, while a wrist device is a wearable that is worn on the wrist. Compared to smartphones, smart wrist devices are physically smaller overall and have a considerably smaller display. Also, the main goal of smart wrist devices is to give users rapid access to features such as monitoring fitness and health data, managing smart home appliances, receiving alerts, etc. Smartphones can do more than just browse the web. They can play media, browse mobile apps, and perform more complex computing tasks. Regarding the input method, a smart wrist device usually lacks the complete touchscreen and virtual keyboard seen on most smartphones, instead relying more on voice commands, touch gestures, and physical buttons for input. Both devices connect to the internet and sync with other devices. However, smart wrist devices often rely more on Bluetooth pairing with a smartphone to access internet connectivity and cloud services. Finally, in terms of use cases, a smart wrist device tends to be better suited for quick interactions, fitness/health tracking, and remote control of smart home/IoT devices. In contrast, smartphones are more versatile for general-purpose computing, communication, and entertainment. Some use cases and/or functionalities have already been integrated into wrist-worn wearable devices for some time, but with the advancement of both scientific and industrial research, manufacturers are gradually increasing the portfolio of available use cases. Figure 1 shows the functionalities already integrated and those in the process of being integrated into smart wrist devices.

An important innovation that has emerged in recent years, and which has led developers and researchers to the use of smart wrist devices for medical purposes, is the direct access to the raw data, extracted openly from the wrist device or the smartphone connected to it. This interest has enabled the development of research strands in various medical fields, such as the evaluation and classification of movement disorders.

The main aim of this review is to provide a collection of the most recent research advancements made in the field of smart wrist devices for monitoring specific movement disorders. This extensive review will help medical staff, caregivers, researchers, and engineers involved in the development of solutions in these research areas, offering a systematic and comprehensive analysis of the most relevant literature works, along with a general idea of recent trends and future developments.

This paper is organized as follows: after this introductory section, Section 2 analyzes in detail the criteria adopted for the selection of the articles in this review, providing additional information on both the wearable smart devices examined and the specific movement disorders for which a correlation with these smart devices was found. In Section 3, the review of the state-of-the-art along the taxonomy of considered movement disorders is presented. Other reviews on movement disorders or the use of smart wrist devices in different contexts are introduced in Section 4. Section 5 describes challenges and open research issues in movement disorder investigations through smart wrist devices. Finally, the conclusion and research directions for future work are presented in Section 6.

## 2. Materials and Methods

An extension of the Preferred Reporting Items for Systematic Reviews and Meta-Analyses (PRISMA) was adopted in this review article as the systematic review methodology [16]. Specifically, here we used PRISMA-S, a 16-item checklist that covers multiple aspects of the search process for systematic reviews [17]. The methodology described by PRISMA-S integrates the PRISMA Statement and its extensions through a checklist that can be used by interdisciplinary authors and peer reviewers to verify that each component of a research paper is fully reported and thus reproducible. A total of three databases were searched, including Scopus, PubMed, and IEEE Xplore Digital Library to identify relevant studies published from 2014 until July 2024.

The search strategy included a compressive combination of keywords and mesh terms related to smartwatches, wristbands, bracelets, and a range of movement disorders. The latter group of terms was declined through the disorders for which the use of smart wrist devices is effective. Search phrases were selected based on the main research question “How are smartwatches or wristbands or bracelets used for movement disorders?”. The search phrases, presented in Table 1, varied slightly between the three databases previously introduced. In the following subsections, in addition to the description of the inclusion and exclusion criteria that contributed to the final screening of the analyzed articles, details are given on the different types of smart wrist devices on the market. Also, a categorization of them is reported to provide as much information as possible for the investigated movement disorders through smart wrist devices.

### 2.1. Overview of Smart Wrist Devices

In recent years, a technological evolution in various sectors was witnessed such as telecommunications, information technology, and home automation. Still, there has also been a great spread of wearable devices, or so-called wearable technology. It brings together all electronic devices that can be worn and that perform various functions. The term ‘smart wrist device’ refers to a category of wrist-worn wearable sensors that are simple to use, and widely popular among the world’s population due to both their low cost and ease of use. Where even a smartphone theoretically belongs to the latter group, one of the clearest examples being smartwatches and smart bands. This type of device has the following advantages: (1) they often replace the traditional watch, (2) they add tracking and physical measurement functions, and (3) they integrate seamlessly with the smartphone. However, smartwatches and smart bands are not the same thing. While the design of the smartwatch may be reminiscent of watches, the integration with smartphones via Bluetooth also allows one to monitor physical or sporting activity, receive notifications on calls, messages, and chats, reply directly from the wrist, take photos or videos if the device has a camera, and control the smartphone remotely. In this respect, smartwatches are true extensions of the smartphone. There are various smartwatch models on the market, varying in features, functionality, and price. Table 2 shows some of the most popular and/or best-selling smartwatches.

About smart bands or fitness trackers, one way of looking at them is to consider such smart wrist devices as depowered smartwatches or rather specialized smartwatches. They are devices designed to fulfill a very specific function, that of supporting sports or physical activity. To do this, they use sensors that measure heart rate, oxygen saturation, specialized training, or gym programs, and IP66, or higher protection against water and dust. In addition, virtually all smart bands connect to the smartphone to send notifications about incoming messages and calls. Unlike the best smartwatches, they do not include GPS modules or SIM slots, which makes them less complete. However, thanks to their more basic and sports-oriented functions, smart bands and fitness trackers are smaller, lighter, and less expensive. Table 3 shows some of the most popular and/or best-selling smart bands currently available on the market.

Smartwatches and smart bands are not the same thing. Although they are part of the same wearable technology sector, they are two different devices that perform different functions. Certainly, suppose the use of the smartwatch or smart band is related to non-daily use but possibly to clinical use. In that case, choosing a device based on specific parameters such as hardware features, certification, or the type of data made available is appropriate.

#### Smart Wrist Device Technology

A wrist device is a system that combines both software and hardware. In addition to providing health management, sports tracking, payment, and other services, it primarily uses a variety of sensors to gather physiological and user activity data. It then uses wireless communication technology to connect to smartphones or other devices to synchronize and push messages, emails, calls, and other information. The distinctive features of wearable technology (specifically smart wrist devices) include their compact size, little intrusiveness, prolonged wearability, and the capacity to track environmental and user factors. Smart wrist devices integrate, for example, inertial motion units (IMUs) like gyroscopes, magnetometers, and accelerometers that measure motion by linear acceleration, angular velocity, and magnetic field variations. Also, most wrist devices (even inexpensive ones) now integrate optical sensors that measure blood flow, skin temperature parameters, ECG, and EMG using surface electrodes. The data processor then uses algorithms and models to process and compute the data collected by the sensor and turn it into actionable information. The user can view the processed data on the data display screen as either text or visuals. Users can choose various functions, examine various information, and interact via a touch screen or knob. Through Bluetooth or Wi-Fi, the integrated communication module can be linked to smartphones or other gadgets to send data. Rechargeable lithium batteries are used to power many of the power supply components. Data acquisition, processing, display, transmission, power supply, and communication modules are all included in smart wrist devices. The main component of the hardware is the processor, which oversees the execution of the watch’s numerous features and apps to maintain regular operation. Applications, system files, and user personal information are all stored in the RAM. Its software platform, or operating system, is in charge of installing and running apps, managing and controlling hardware, and communicating with interfaces. To show time, information, sports data, and other data, the display panel typically uses an LCD or OLED screen. To drive and control the screen, the display screen has an LED drive and boost circuit. Integrated acceleration. The technology that makes it possible to provide accurate and real-time physiological data through any kind of smart wrist device has a greater influence on our day-to-day activities. However, it is important to note that there are several challenges with using a wrist device as a diagnostic instrument, such as connectivity, power consumption, and obtaining and maintaining accuracy. Their manufacture is challenging since it calls for the integration of several sensors and miniaturization. These remotely monitored tailored diagnostic techniques raise questions about data protection, sharing, and storage when compared to traditional diagnostic techniques. In this context, regulatory organizations are crucial.

### 2.2. Movement Disorders Categorization

**Figure 2:**
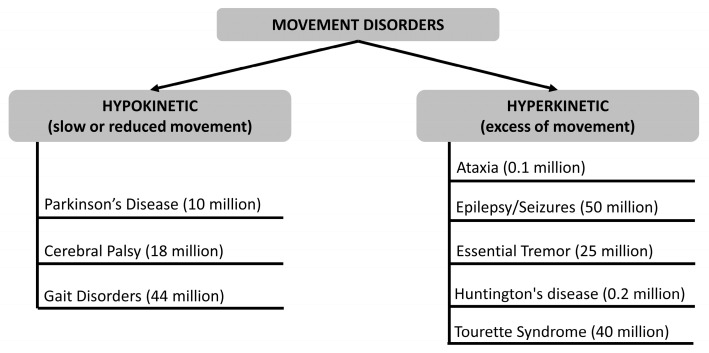
Classification of movement disorders with an approximate indication of worldwide incidence.

As evidenced by numerous publications, hypokinetic and hyperkinetic disorders are among the diverse range of neurological conditions known as movement disorders. Specifically, hypokinetic movement disorders are characterized by reduced movements, such as rigidity and akinesia/bradykinesia. In contrast, hyperkinetic movement disorders are characterized by excessive movements and include many motor manifestations. This sub-section aims to provide a brief clinical description of hypokinetic and hyperkinetic movement disorders (schematically shown in Figure 2) for which information can be provided through the use of wearable smart wrist sensors and for which there is at least one article downstream of the exclusion and inclusion criteria adopted in this review (see Section 2.3), i.e., ataxia, epilepsy, essential tremor, gait disorders, Parkinson’s disease (PD), Huntington’s disease (HD), seizure detection, cerebral palsy (CP), or unilateral CP (UCP), and Tourette syndrome.

#### 2.2.1. Hypokinetic Movement Disorders

PD is a classic hypokinetic movement disorder and one of the most common and widely recognized neurodegenerative conditions. PD symptoms start slowly and get worse over time. Because the rest tremor is so evident, it is frequently the first symptom that the patient notices. However, bradykinesia might occasionally be the first symptom of the condition, and tremors may never emerge in some patients. Bradykinesia is characterized by slowness, including smaller and slower handwriting, a shorter stride and arm swing when walking, a lowered facial expression, and a reduced voice amplitude. Initially, rest tremors may be sporadic, occurring mainly during stressful times; subsequently, they become more consistent and increase in intensity during stressful or exciting events. The symptoms gradually get worse over time, and if left untreated, they can cause severe immobility and falls. While PD can strike at any age, older people are the most likely to experience its start, with a peak age of 60 years or so. With a lifetime risk of roughly 2%, the chance of having PD rises with age [18].

CP is a term used to describe a group of neurological disorders that manifest in infancy or early childhood and result in permanent alterations to body movement and muscle coordination. The underlying cause of CP is damage to or abnormalities within the developing brain, which disrupts the brain’s capacity to regulate movement and maintain posture and balance. The term ‘cerebral’ refers to the brain, while ‘palsy’ denotes the loss or impairment of motor function. UCP is the most prevalent form of CP, accounting for more than 30% of all children with a diagnosis. One side of the body experiences a severe loss of muscular function in a newborn with UCP. Both sides are affected, although the left side of the body will be more severely affected if the right side of the brain is destroyed. These disabilities have a long-term impact on the child’s social skills because they force them to devote more of their energy to completing basic activities rather than developing social skills. Due to superior coordination on one side, children with unilateral cerebral palsy will acquire a favored side sooner than healthy children. This covers which foot to place weight on and which hand is dominant. Several parents observe their children crawling unbalanced before they can walk [19].

The motion made while a subject runs or walks is generally referred to as “gait”. Walking is an intricate series of movements that involves cooperation between the heart and lungs as well as the brain, bones, and muscles. Should an issue arise with any of those systems, it may have an impact on the gait. The term for referring to this is “gait disorder”. Disorders of the gait may be a sign of something more serious. They are more prevalent in older people. A gait disorder can make a subject more likely to fall and hurt himself, which can lower his quality of life. Over the years, different types of gait disorders have been classified. Some of the more common gait disorders include the following: neuropathic, myopathic (also known as waddling gait), hemiplegic, diplegic, parkinsonian, and ataxic. There are other reasons why a subject might find it hard to walk. Walking might be impacted by chronic pain from diseases like arthritis or injuries from the past. Feet weakness or pain could also be a factor. Also, it is frequent to experience difficulties with balance due to a movement-related inner ear disorder [20,21].

#### 2.2.2. Hyperkinetic Movement Disorders

The term ataxia means ‘without coordination’. This movement disorder causes a loss of motor control in the arms and legs. This results in walking difficulties and a lack of balance and coordination. Fingers, hands, arms, legs, trunk, voice, and even eye movements can be affected by ataxia. It can result from several factors, such as nerve damage, tumors, stroke, and multiple sclerosis. The kind of ataxia may have different symptoms and start times. Usually, the most typical are balance and coordination problems, inability to coordinate hands, arms, and legs, speech slurring, broad-based gait, and having trouble writing and eating. The aforementioned disorder is generally diagnosed with genetic testing, magnetic resonance imaging (MRI), or lab tests including blood and urine studies. Furthermore, hereditary ataxia cannot be cured and there is no medication to address the ataxia symptoms [22].

Epilepsy is a neurological condition involving the brain that makes people more susceptible to having recurrent unprovoked seizures. It is one of the most widespread nervous system disorders, affecting individuals of all ages, races, and ethnicities. Epilepsy may result from a variety of factors, such as an imbalance in the chemicals called neurotransmitters that communicate nerves, tumors, strokes, and damage to the brain from disease or trauma, alone or in combination. Most of the time, epilepsy may have no known etiology. Although epilepsy cannot be cured, many treatment options are available. Up to 70% of people with epilepsy can manage the disease with medications. Treatments to control epilepsy also include special diets (usually in addition to anti-seizure medications) and surgery [23].

A seizure takes place when a portion of the brain experiences an abrupt spike in aberrant electrical signals that momentarily disrupts regular brain electrical activity. The region and extent of the brain affected, as well as the events that transpire during the seizure, determine the type of seizure. Generalized seizures (absence, atonic, tonic-clonic, myoclonic) and partial seizures (simple and complex) are the two main types of epileptic seizures. Various distinct kinds of seizures fall under these categories: focal, atonic, myoclonic, and febrile seizures. Even once symptoms start, it may take some time to fully comprehend the magnitude of the seizure; nevertheless, diagnostic testing and a thorough medical assessment can help. Diagnostic tests and a physical examination are used to diagnose seizures. During the examination, the doctor asks when the seizures started and gets the child’s and family’s whole medical history. Neurological issues may be the cause of seizures, necessitating additional medical monitoring [24].

Essential tremor is a disorder of neurological origin that evolves progressively. It is a movement disorder, characterized by a rhythmic movement or tremor of the hands, head, trunk, legs, or voice. There are two types of essential tremors: kinetic and postural. Essential tremor is not life-threatening, but life-altering. Thus, sufferers lose the ability to perform simple daily actions that they used to perform normally, e.g., working, or driving a car. The main characteristic of essential tremor is a rhythmic tremor, which occurs when the patient makes voluntary movements, or even when trying to maintain a suspended position, or against gravity. Most people with essential tremor suffer from both postural and kinetic tremors. Today, there are still no treatments that can control the development of essential tremor or that can cure it. The goals of treating essential tremor will be to reduce the severity of the symptoms and improve the functioning of the patient’s locomotor system [25].

HD is a hereditary disorder that causes progressive degeneration of neurons in specific areas of the brain (particularly the striatal nucleus and the cognitive cortex). Sufferers begin to show typical symptoms from the age of 30–40 years, but sometimes a ‘juvenile’ form of the disease can appear as early as the second decade of life. HD is an autosomal dominant genetic disease: this means that an affected parent has a 50% chance of passing it on to their children. The symptoms are related to the degeneration of the nervous tissue that is no longer able to transmit stimuli. In particular, the symptoms that manifest themselves in the motor system are jerky and uncoordinated movements often accompanied by tremors; muscle problems, such as contractures and stiffness of muscles (dystonia); slowed or abnormal eye movements; inability to maintain balance and posture; and inability to process speech. Unfortunately, there is currently no decisive cure for this condition. However, the main symptoms, both cognitive and motor, can be alleviated with certain medications that improve the patient’s quality of life and help prevent complications [26].

Tourette Syndrome is a neurodevelopmental disorder that typically arises during childhood or adolescence and is characterized by inducing motor and phonic (or vocal) tics. The precise cause of Tourette syndrome is unknown. On the subject, however, some theories consider the intervention of genetic factors, neurological factors, and/or environmental factors. The tics that characterize Tourette’s syndrome consist of repeated, involuntary movements and sounds; they are also difficult to control and completely unmotivated, i.e., without a specific purpose. The tics of Tourette syndrome are exhausting, repeated many times during the day, and long-lasting (more than a year). In most patients with Tourette’s syndrome, the tics disappear or subside considerably by the time they reach adulthood. After adolescence, very few people continue to present, with the same frequency, disorders typical of Tourette syndrome. Currently, there is no specific test or instrumental examination that allows for the diagnosis of Tourette syndrome. As with the diagnosis, there is no specific therapy for Tourette’s syndrome. However, it is possible to alleviate symptoms through medication and psychotherapy; these treatments improve the quality of life of young patients, particularly when they suffer from severe forms of the disorder [27].

### 2.3. Article Selection, Inclusion and Exclusion Criteria

The queries in Table 1 returned a total of 911 articles (429 from Scopus, 437 from PubMed, and 45 from the IEEE Xplore Digital Library). Only articles produced within the last 10 years, starting from January 2014, were selected. In the screening phase, 351 duplicates were first eliminated and then the remaining articles (560) were analyzed by title and abstract, after checking the availability of the full text. Then, PDF copies of all remaining articles were downloaded. The eligibility criteria for inclusion in the review were as follows:

- Articles published in an indexed journal (conference abstracts, workshop results, preprint articles, and posters were not considered for inclusion in the review).
- Articles in which a smart wrist device is used (both commercial and prototype).
- Articles that present results from studies where data were collected using humans.

On the other hand, the eligibility criteria for exclusion in the review were as follows:

- Articles in which the device used for the assessment of the movement disorder is not wrist-worn.
- Articles that do not provide information on movement disorders (these are listed in Section 2.2).
- Articles containing reviews or surveys.
- Articles not produced in the English language.
- Articles downloadable only against payment.

**Figure 3:**
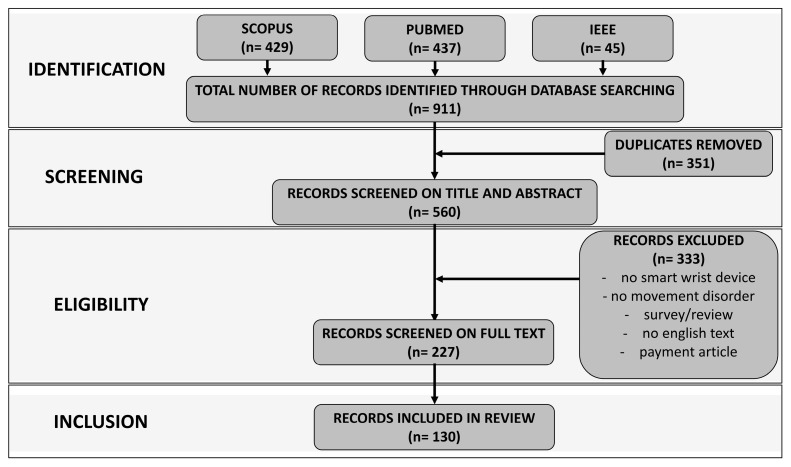
Flow diagram generated with PRISMA-S methodology, depicting the reviewers’ process of finding published data on the considered topic and how they decided whether to include it in the review.

Following the definition of inclusion and exclusion criteria, 227 articles remained to be screened. A more in-depth reading was necessary for these articles. Specifically, the internal manuscript was analyzed to include those articles that in some way classified movement disorders using the raw data provided by the wrist device. In fact, through this in-depth process, articles were excluded in which data extracted from the smart wrist device was used but which did not provide information on movement, such as heart rate, oxygenation, number of steps, or sleep stages. Ultimately, the articles that met the inclusion criteria, and therefore, were considered for our proposed comprehensive study, were 130. The study selection process is depicted in Figure 3.

**Figure 4:**
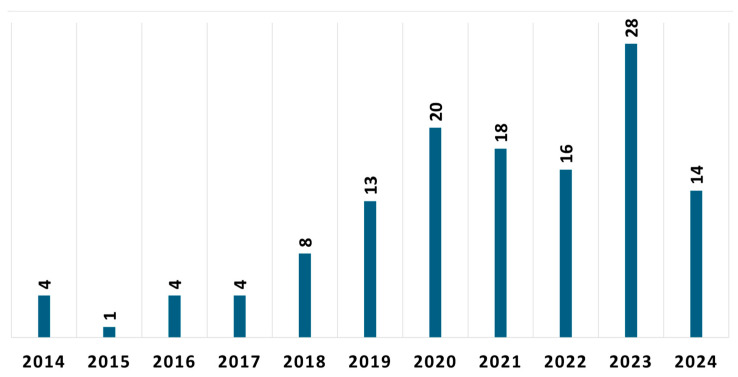
Distribution of the articles by year of publication.

On the other hand, Figure 4 illustrates the number of publications on movement disorders and smart wrist devices over the period January 2014–July 2024. It is noteworthy that limited articles have been released until 2017. The number of publications started to rise in 2019 significantly, and by 2020, 96 publications highlighting the use of smart wrist devices to analyze movement disorders had been found. This indicated a notable rise in interest which is certainly related to the COVID-19 pandemic during which the world’s population discovered a new way of living and monitoring their health, including through commercial devices never previously used for this purpose. The trend shows a decrease in publications in 2024, due solely to the fact that the literature search for articles ended in July.

**Figure 5:**
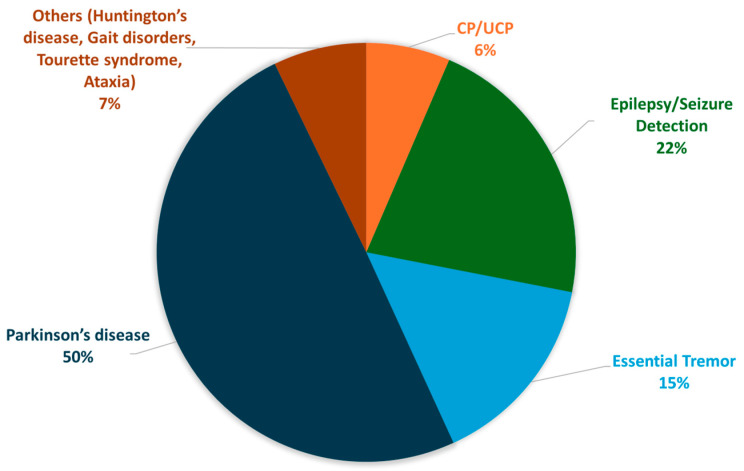
Distribution of the articles by movement disorder.

Most selected articles (sixty-eight of the one hundred thirty publications included in the review) addressed using smart wrist devices for PD. The topic of thirty publications was epilepsy/seizure detection with smart wrist devices. The essential tremor and the CP/UCP were the subjects of thirteen and eleven publications, respectively. The remaining nine publications cover a variety of movement disorders which, however, are less investigated by the scientific community, including gait disorders (four publications), HD (three publications), ataxia (one publication), and Tourette syndrome (one publication). Seven articles also describe the use of smart wrist devices concerning two movement disorders: PD and essential tremor. The distribution of publications by movement disorder is shown in Figure 5.

## 3. Scientific Articles on Movement Disorders

This section of the review article aims to describe the contents of each article briefly included downstream of the selection and inclusion criteria illustrated in Figure 3, categorizing them according to the movement disorder(s) treated. At the end of each subsection, there is a table with some summary information on each article, such as the type (commercial or non-commercial) of smart wrist device used, the number of end-users involved in the validation/experimentation if any, and possible availability of datasets.

### 3.1. Parkinson’s Disease

**Figure 6:**
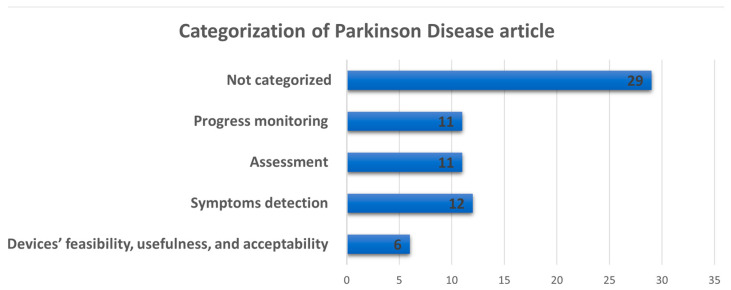
Categorization of the articles related to PD movement disorder.

PD is the movement disorder most investigated in the last 10 years by the scientific community, using smart wrist devices. For this reason, this section includes many articles; consequently, it was deemed appropriate to divide them into thematic subsections, considering different aspects of diseases. The distribution of these articles is depicted in Figure 6.

#### 3.1.1. Articles Dealing with Devices’ Feasibility, Usefulness, and Acceptability Used for PD Evaluation

Firstly, the feasibility, usefulness, and acceptability of the device/technology proposed and designed for the assessment of Parkinson’s have been described in a series of articles, these features being essential for large-scale use [28,29,30,31,32,33].

The study proposed in [28] examines the possibility of using a novel Digital Health Technology System (DHTS) that integrates a smartwatch, smartphone, and IMU to track mobility and evaluate people with Parkinson’s adherence to medication. In particular, the IMU makes it possible to monitor digital mobility outcomes continuously; the smartwatch interacts with a digital screen to record self-reported medication intakes and remind users when to take their medications; the smartphone notifies the smartwatch and captures contextual data. Participants also keep a daily journal in which they document any motor complications. The work concludes that the DHTS was well tolerated concerning usability and that the latter is closely related to the age of the end-user, but further work is necessary to determine whether the implemented DHTS can be implemented for clinical decision-making. Also, another research supports the potential of gathering data from PD patients remotely [29]. PD participants used an app to report symptoms and medication intake in addition to streaming data from a smartwatch. However, participants frequently skipped days, and after the first three months, there was a gradual decrease in the number of reports and streaming, concluding that there were probably several contributing factors to this decline in compliance, such as study fatigue, a decline in the study of technology’s novelty, problems unique to the device, and technical constraints. Morgan et al. [30] introduced a small-scale feasibility and acceptability study in which 12 pairs of participants (one with PD and one healthy control) stayed and lived independently in a home-like setting for five days. The home was equipped with different technologies which included environmental monitoring, camera sensors, wrist-worn accelerometry, and appliance monitoring. Together with participant diaries, clinical rating scales, and professional clinician annotations of color video images, these data were also gathered, and machine learning (ML) was used to look for a signal to discriminate between PD and control, and between PD symptoms ‘on’ and ‘off’ medications. The potential of collecting clinically significant data using a technology-based platform that consists of a smartphone, a smartwatch, and two smart insoles was investigated in [31], concluding that 87% of participants finished the protocol and according to regression analysis, careers’ burden was the primary factor linked to high use. The authors also highlighted that how the system is used was influenced by patients’ self-rated health status and motor aspects of daily living experiences. The research described in [32] evaluated the practicality of employing a wearable platform for extensive data collection among a substantial cohort of patients with PD. The authors focused on recruitment success, attrition rates, compliance, and system usability, showing that it was feasible to deploy a technology platform consisting of consumer-grade wearable and mobile devices for long-term data collection in a large and geographically diverse PD population. Liikkanen et al. [33] conducted a pilot study, utilizing a consumer-grade accelerometer and a mobile application to track symptoms associated with PD. Analysis of data revealed that the state of movement can influence the manifestation of PD symptoms. Furthermore, their findings indicated that unsupervised variational autoencoders facilitate estimating movement states with relative ease. The works discussed in this section are summarized in Table 4.

#### 3.1.2. Articles Dealing with PD Symptom Detection Through Smart Wrist Devices

Another wide range of articles illustrates how smart wrist devices can be used to detect PD or symptoms associated with it [34,35,36,37,38,39,40,41,42,43,44,45]. For example, in [34] the possibility of using a wearable sensor that records autonomic nervous system activity predicting wearing-off in people on L-dopa was investigated. In the proposed work, there were PD subjects on L-dopa who recorded a diary of their on/off status over 24 h while wearing a commercial wearable sensor. The obtained results suggested that autonomic nervous system dynamics can be used to assess the on/off phenomenon in people with PD taking L-dopa but must be individually calibrated. The authors of [35] presented a method to objectively evaluate bradykinesia and tremor in ten older adults with PD who were over 60 years old and had bradykinesia, as well as twenty older adults in good health who were over 60 years old, capturing physical movements with a bracelet that was created with inertial sensors—a 3D accelerometer and gyroscope. Temporal and spectral features were extracted to distinguish the patients from healthy controls. Non-linear temporal and spectral features exhibited the largest differences, obtaining an accuracy of 91.7% with the K-Nearest Neighbors (KNN) classifier in detecting subjects. In a clinical setting, the device helps the physician diagnose patients’ bradykinesia and tremor more rapidly and accurately. Also, the findings of another very recent study suggested that bradykinesia severity can be detected and assessed using commercial smartwatches in conjunction with artificial intelligence techniques. Additionally, utilizing data from a single tri-axial gyroscope produced the best results; combining data from angular velocity and acceleration did not result in any additional gains. Moreover, the limited amount of data used in Deep Learning (DL) methods led to their poor performance when compared to traditional ML-based classification approaches [36]. The work proposed in [37] aimed to develop a DL approach based on wearable devices that can more reliably and conveniently identify PD hand tremors in everyday situations. Initially, the wrist acceleration signals were intended to be collected by the inertial sensor system with a tri-axial accelerometer. Then, a nine-layer Convolutional Neural Network (CNN) architecture was created to detect PD hand tremors. Ultimately, the experiment demonstrated the efficacy of this method by comparing it to standard ML classifiers, achieving a classification accuracy of about 97.1%. A sensor for detecting and monitoring Parkinson’s disease was explored in [38]. The authors analyzed the UK Biobank dataset, which consisted of one week of wrist-worn accelerometry from a population with PD and an age-matched healthy control population. From the automatically segmented data, measures of movement dispersion and dimensionality were extracted. ML classifiers identified PD with an AUC of 0.69 on gait data, 0.84 on low-movement data, and 0.85 on a combination of both. Brink-Kjær et al. [39] developed a walking bout detection algorithm based on tri-axial accelerometer data obtained passively through wrist actigraphy. The results showed that a gait detection model could identify walking bouts with high precision. This experiment is the first step in investigating movement characteristics associated with Parkinsonism. In patients with RBD, this analysis could indicate an elevated risk of PD progression. The four main objectives of the study reported in [40] were to use a wrist accelerometer to detect PD early in a free-living setting, to examine the effects of two feature engineering techniques on the performance of classification models, to examine the effects of various windowing strategies and the features that go along with them on the models’ performance, and to examine the effects of data volume on the performance of classification models. According to another very recent research, DL can be used autonomously and at high temporal resolution to extract expert-level information about the motor state of people with Parkinson’s disease (PwP) from a single wrist sensor in free-living scenarios. In as little as one-minute time windows, clinically significant trends and changes in motor state can be identified with high accuracy thanks to probabilistic data and smoothing techniques. Additionally, in the same article, it was demonstrated that the proposed approach can identify each patient’s unique motor state regardless of the activities they engage in an uncontrolled, free-living environment, and it can be applied to the whole population of PwP based on data from patients who have never been seen before [41]. In [42], the authors used wrist-worn accelerometer data from laboratories and field studies to analyze various algorithms and features for PD tremor detection. Specifically, they have found several feature sets, such as a tremor spectrum extraction method, that showed improvement over earlier work. The authors found that a CNN trained on tremor spectra yielded the most effective results in data classification. Reference [43] describes algorithms for extracting gait sequences (GSs) of people with Parkinson’s disease (PwP) using a wrist-worn sensor. The performance for detecting GSs as regions of interest for further gait parameter extraction and quantification of gait duration is lower than for the lower back position. However, wrist-worn sensors may justify a certain level of lower gait quantification performance. In another work, thirteen PD patients wore a commercial smartwatch that collected tri-axial accelerometer data and a flexible, skin-mounted sensor that collected gyroscope and tri-axial accelerometer data on their primarily affected hand. A clinician graded the degree of bradykinesia and tremor in each limb as the participants completed a battery of standardized motor tasks. Tremor and bradykinesia were classified using ML models trained on scored data. The model’s performance was compared with and without different kinds of sensors, data sampling rates, and pre-engineered feature categories [44]. The aim of [45] was to detect Freezing of Gait (FoG), a motor impairment among patients with advanced PD. Here, research using data from 11 patients with FoG and PD reveals certain characteristics of wrist movements, such as power in various frequency ranges and statistical information from rotation and acceleration data, associated with gait freeze. Additionally, the authors concluded that FoG can be detected with high accuracy (of about 90%) using wrist motion and specific ML models. The works discussed in this section are summarized in Table 5.

#### 3.1.3. Articles Dealing with PD Assessment Using Smart Wrist Devices

PD assessment is also a research topic covered in numerous articles in recent years [46,47,48,49,50,51,52,53,54,55,56]. Battista et al. [46,47] investigated in two different works the possible relationship between the Unified Parkinson’s Disease Rating Scale (UPRDS) scores and the information provided by the novel commercial tool “PD-watch”. Also, the authors explored the agreement between the continuous data generated by the proposed tool method and data reported in the patient diaries. Although the PD-watch might make it possible to monitor some motor aspects continuously and objectively, no information about non-motor aspects is offered. On the other hand, continuous and long-term monitoring of movement disorders for 24 h a day, rather than just during the brief clinical examination period, may offer supplemental information to the UPDRS [48] to assess the analytical and clinical validity of multi-sensor smartwatch measurements in hospitalized and home-based settings using a twice-daily virtual motor examination (VME) at times representing medication OFF/ON states, useful for tracking disease progression and treatment response in PD. The obtained results showed that unsupervised digital measurements of motor features with wrist-worn sensors were sensitive to medication state and are reliable in naturalistic settings. Another article [49] examined the potential applications of meal microstructure and eating behavior as objective markers of PD. This was accomplished by counting the upward hand movements that take place prior to each food intake moment during a meal. Using a Support Vector Machine (SVM) array, the method’s initial phase deals with the recognition of wrist micromovements that happen during a meal. Additionally, a Recurrent Neural Network (RNN) with two Long-Short-Term Memory (LSTM) cells can detect the bite moments of the meal by simulating the temporal evolution of the recognized wrist micromovements. Experimental results using three datasets (one in clinical and two in-the-wild settings) revealed the high potential of the proposed approach towards the classification of in-meal eating profiles to the PD. The study proposed in [50] examined the viability of utilizing DL techniques in conjunction with consumer smartwatches that have not been altered to offer PD patients a low-cost, self-contained option for the automatic detection and evaluation of the magnitude and constancy of their resting tremor. Based on the obtained results, it can be concluded that the suggested methods, which utilize CNN with numerous outputs, may accurately determine the magnitude and consistency of resting tremors by analyzing the data gathered while executing a series of pre-planned activities and rest intervals. A novel wrist device was presented in [51], the Parkinson’s KinetiGraph which consists of a small device worn on the wrist for collecting data over 6–10 days providing a report for the doctor that shows how motor symptoms and complications of PD (such as slowness of movement, stiffness, tremor, and dyskinesias) vary throughout the day. The authors used a Markow model as the methodology for clinical assessment and the results obtained were generally consistent across a range of sensitivity analyses. The authors of the study reported in [52] showed that two sensors—one on the upper extremity (hand), and one on the lower extremity (ankle or thigh), of the most affected side of the body, could reasonably accurately estimate the severity of total body dyskinesia that occurs during routine purposeful activities. A novel motor assessment system was successfully proposed in [53], where sixteen PD patients employed a smartphone and smartwatch-based monitoring system to collect objective data on motor symptoms one week before and four weeks after a doctor’s suggested therapeutic modification. The participants returned to the clinic after five weeks to discuss their findings with their physician, who then recommended therapy based on the reports and his clinical assessment. The medication diary and the symptom scores were synchronized, and weekly and hourly timelines were used to compute the therapy’s temporal effects. Works [54,55] describe the obtained results in a multicenter study, where a smartphone research application and a commercially available smartwatch were used to record important motor and non-motor characteristics of early, untreated PD. These measurements showed varying connections with established measures, differed from age-matched controls, and hold the potential to be objective, practical assessments of the illness for use in future research. In [56], the performance and usability of a novel wearable device to aid in identifying Parkinsonian motor disorders are evaluated. The methodology proposed is founded upon the utilization of a wrist-worn measuring device, a passive, continuous recording session, and the calculation of two digital biomarkers: motor activity and rest tremor index. A second phase is proposed, based on the execution of some motor tests, with the objective of confirming the results of the passive recording. The findings of the study demonstrated that: (a) There was no significant difference in motor activity between controls and PD patients with mild-to-moderate tremors at rest, whereas it was higher in PD patients with modest tremors; (b) The tremor index was smaller in controls than in PD patients with mild-to-moderate tremors at rest, and there was no significant difference between controls and PD patients with slight tremors at rest; (c) The combination of the aforementioned two motor parameters improved the ability to differentiate between controls and PD patients. The works discussed in this section are summarized in Table 6.

#### 3.1.4. Articles Investigating PD Progress Monitoring Using Smart Wrist Devices

Monitoring the progress of PD is also the subject of interesting research articles [57,58,59,60,61,62,63,64,65,66,67]. By combining DL-assisted data analysis with designed flexible triboelectric sensors (FTES), the authors of [57] created an intelligent Internet of Medical Things (IoMT) system that serves as a platform for healthcare that allows for individualized monitoring and treatment for PD patients and the elderly. The IoMT system achieved various functions including motion state determination with a classification accuracy of 97.3%. This work concluded that the developed IoMT based on the FTES provides an important reference for clinicians and healthcare practitioners in developing treatment plans for PD patients. Channa et al. [58] presented a novel method for evaluating and tracking people with PD using an eHealth platform. The focal point of this platform is an intelligent and non-commercial wristband always linked to the cloud. The most affected limb’s wrist is the one where the wristband gathers motion data and sends it over Wi-Fi to a cloud-based platform as a service. Here, the data are automatically processed and analyzed to provide real-time feedback on the degree of bradykinesia and tremor in both ON and OFF patients. Moreover, this research added to the objective evaluation of severity, especially after surgical procedures or physical therapy activities. One of its main advantages is the introduced smart-bracelet solution’s architecture, which ideally balances power consumption, network coverage, data transfer rate, and cost. Another interesting paper investigated the development of a novel wearable solution for PD patients that records accelerations and angular velocities from the wrist and ankle (on the most affected side) for prolonged periods, along with wearer triggered PhotoPlethysmoGraphy (PPG) and electrocardiogram (ECG) signals from the palm of the less affected hand. While the inertial data obtained may eventually be utilized to predict gait speed, sleep-wake cycles, and primary motor symptoms of PD, PPG, and ECG signals may be used to measure blood pressure without the need for a cuff. By helping doctors distinguish between symptoms caused by hypotension and Levodopa insufficiency, this information may enhance patient safety and drug decision-making [59]. In [60], to capture individual variances, the authors attempted to define personalized motor fluctuation profiles. For two weeks, 21 advanced PD patients with movement fluctuations were tracked using a commercial smartwatch and a smartphone app (Intel Pharma Analytics Platform). The smartwatch continually recorded active data, such as timed up and go, finger tapping, hand tremor, and hand rotation performed once in OFF and once in ON levodopa periods, as well as passive data, such as tremor, dyskinesia, and level of activity using specific algorithms. From the results, the authors found notable distinctions between the distinct symptom patterns and levodopa-related alterations throughout the home-based monitoring, which enabled the classification of the subjects into four profiles based on data-driven motor fluctuations. A novel framework named MONIPAR was introduced in [61], able to monitor motor symptoms such as resting tremors and bradykinesia.

This architectural approach was operationalized through the creation of the ad hoc, which gathered motion signals while standard exercises were being performed using a commercially available smartwatch. The correlation analysis, conducted using the data collected by MONIPAR, demonstrated moderate-to-strong correlations between several indicators and two specific MDS-UPDRS exercises, designed to evaluate resting tremors and bradykinesia. In conclusion, the results of this study indicate that MONIPAR has the potential to be employed as an additional tool for the acquisition of information and the monitoring of motor disorders associated with PD, particularly in patients who are in the early stages of the disease. A cross-sectional study was conducted in [62] to test the efficacy of a wrist-worn device in conjunction with machine learning to detect circadian rhythms of sleep, motor, and autonomic disruption. This approach may prove suitable for the objective and non-invasive evaluation of PD patients. An ambulatory circadian monitoring device was used to record the following parameters continuously for seven days: wrist skin temperature, motor acceleration, time spent in movement, hand position, light exposure, and sleep rhythms. The results demonstrated that the best indexes for objectively characterizing the most common symptoms of PD were daytime acceleration (indicative of motor impairment), time in movement during sleep (representative of fragmented sleep), and their ratio. These indexes allowed for a reliable and easy scoring method to evaluate patients. Habets et al. [63] demonstrated that a single wrist-worn accelerometer could be used to successfully classify spontaneous bradykinesia fluctuations at the single-minute time scale when trained on individual and group data in PD patients using ML models. One accelerometer component, the coefficient of variation, was found to be predictive of bradykinesia at the group level over longer durations (e.g., an hour). The categorization of group-trained models was refined through the extension of brief accelerometer time epochs and the augmentation of the number of training patients. For patients with Parkinson’s disease undergoing medication and stimulation therapy, real-time and dynamic monitoring may facilitate precise and personalized therapeutic optimization. In [64], a mobile application has been developed for the assessment of speech, tremor, gait, balance, bradykinesia, and bradyphrenia. The system comprises ten live tests for smartphones and routine passive monitoring using a smartwatch and smartphone. To conduct passive monitoring, 316 participants in the early stages of Parkinson’s disease completed daily activity tests at home and carried a smartphone and smartwatch with them throughout the day. In [65], the authors designed and developed an ambulatory monitoring system that employed smartwatch inertial sensors to track fluctuations in resting tremor and dyskinesia on a continuous basis. The monitoring system demonstrated the capacity to capture symptom changes in response to treatment that aligned with the expectations of the treating clinician in 94% of evaluated subjects. In the remaining 6% of cases, the retrieval of symptom data from the novel ambulatory system identified opportunities for clinically applicable changes in pharmacologic strategy.

In contrast, the primary objective of [66] was to ascertain the feasibility of conducting a large-scale study utilizing a wristband to assess the risk of falls in individuals with PD. The findings substantiated the efficacy of the smart wrist device in discerning gait patterns indicative of an increased risk of falling or near falling. Additionally, the authors posited that the device could be employed to categorize the risk level in frail patients at elevated susceptibility to fracture. A work widely cited by the scientific community detailed the creation of a unique method for detecting the disease stage of PD using algorithms based on data gathered in an unsupervised home setting [67]. Here, the authors assessed if patient-completed symptom diaries and clinician assessments of the illness state could be replicated using this method. The results showed that the specificity for dyskinesia detection in the clinical environment was remarkably high (99%), and home-derived data likewise showed good specificity (93%), although with modest sensitivity (38%). The works discussed in this section are summarized in Table 7.

#### 3.1.5. Other Works That Correlate PD and Smart Wrist Devices

Numerous articles correlating the use of smart wrist devices with PD have also been published in the last 10 years, and not in the categories previously discussed in this section. In [68], a passive energy sink was designed to help individuals with natural tremors or PD with their elbow and wrist tremors. Instead of using a pair of springs and dampers, the suggested system used a single-shape memory alloy (SMA) spring, reducing the number of moving components in the absorber by half. Real-world tremor data and harmonic excitations were used in the simulations. According to the results, the SMA absorber outperformed the viscous absorber in lowering the resonance amplitude. Additionally, in the 3–25 Hz range, the SMA absorber dramatically reduced the tremor power under random excitations compared to the dense absorber.

Varghese et al. [69] delineated the methodology for a novel Smart Device System (SDS) for multi-modal high-resolution acceleration measurement of patients with PD or essential tremor within a clinical setting. A two-year prospective observational study was conducted with the objective of identifying new phenotypical biomarkers and training an artificial intelligence system. The SDS was integrated and tested within a 20-minute assessment comprising smartphone-based questionnaires, two smartwatches on both wrists, and tablet-based Archimedean spiral drawings for deeper tremor analyses. In [70], a novel platform, designated “PD Manager mHealth”, was introduced. The objective of the platform was to facilitate the continuous provision of symptom data, thereby enhancing clinical comprehension of each patient’s status and guiding treatment planning. The trial was designed to evaluate two aspects: the study aimed to gain insight into two key areas: (1) patient (and family caregiver) perspectives regarding comfort, acceptability, and ease of use; and (2) clinician perspectives regarding the system’s acceptability in clinical practice and the usefulness of the data generated by the platform for clinical decision-making. The works [71,72] both described specific deep brain stimulation (DBS) programming for PD. Specifically, in the pilot study proposed in [71], the authors presented and validated a method for automated parameter selection in deep brain stimulation (DBS) for the treatment of tremors in patients with Parkinson’s disease (PD). In comparison to chronic clinical DBS settings chosen by skilled clinician programmers, the proposed automated programming approach was able to identify DBS settings that controlled tremors at least as well. Furthermore, the reported findings demonstrated that precision IMU data can offer a more precise assessment of tremor severity than the rather coarse, integer-based MDS-UPDRS clinical scale, and may be utilized as a substitute for clinical evaluations. In contrast, the authors of [72] described and evaluated a patient-specific, automated, closed-loop framework for DBS programming that used a smartwatch to measure tremor and automatically modify DBS parameters based on suggestions from a closed-loop optimization algorithm, thereby eliminating the necessity for a specialized clinician. In this instance, Bayesian optimization, which is a sample-efficient global optimization method, was employed as the foundation of the DBS programming framework, enabling the adaptive learning of each patient’s response to DBS and the suggestion of the subsequent optimal settings for evaluation. The results demonstrated that a fully automated DBS programming framework for the treatment of tremors is both efficient and safe, while offering outcomes that are comparable to those attained by skilled therapists. In [73], the frequency and time-domain tremors of 18 PD patients’ fingers and wrists were examined through a non-commercial wearable device designed to suppress tremors. The quantifiable features of Parkinsonian hand tremors were found to be the flexor/extensor activation ratio, linear acceleration, angular velocity, and angular displacement. The results demonstrated that PD hand tremors exhibited a common multiple-harmonic pattern, with the frequencies of the harmonics forming an arithmetic progression. Further analysis revealed that the second harmonic plays a significant role in the tremor, although not as dominant as the first harmonic. Consequently, it cannot be disregarded. The objective of the authors of [74] was to gain insight into patient compliance in digital trials of two such pathologies, namely Parkinson’s disease (PD) and Huntington’s disease (HD). Compliance was assessed in two remote, six-month clinical trials. The results demonstrated that both studies sustained high compliance levels throughout the six-month duration. During the initial stages of enrollment, notable fluctuations in compliance rates were observed in both groups. It is noteworthy that the daily patterns of smartwatch data streaming were comparable, with a peak at noon and a significant decline at 8 p.m., with a mean of 8.6 h for the PD study and 10.5 h for the HD trial. Pairwise correlation analysis revealed that individual patients exhibited either high or low compliance across all compliance parameters. It is encouraging to see that established timetables and smartphone apps worked as planned to remind people to report their prescription intake and regulate motor tasks at home.

The development of a low-cost wearable bracelet for sufferers of PD and essential tremors able to target both rest and active tremors was detailed in [75]. Using real-world recorded tremor spectrums as input signals, a dynamic vibration absorber was designed. A novel multiple-slot architecture made it possible to suppress several frequencies between 3 and 8 Hz. The upper limb’s response was simulated using finite element analysis both with and without the bracelet. According to the results, tremors could be reduced by up to 68% when suppression was applied generally and up to 98.2% when suppression was applied specifically. Also, the research reported in [76] has mostly concentrated on motor symptoms of PD paying little attention to the disease’s non-motor components. To address this, the authors merged digital multi-sensor data from the Verily Study Watch and longitudinal clinical non-motor evaluation data for 149 individuals with PD diagnoses from the Parkinson’s Progression Monitoring Initiative (PPMI) cohort. They demonstrated a substantial relationship between clinical non-motor assessments of cognitive, autonomic, and daily living impairment and digitally obtained physical activity and sleep measurements. They concluded that to monitor non-motor symptoms, better-targeted digital outcome measures are necessary, as seen by the low predictive performance they saw. Mammen et al. [77] conducted a study designed as a follow-up to the parent WATCH-PD study [54,55]. This was the inaugural study to meticulously assess the prevalence, personal significance, and comparatively bothersome nature of symptoms and impacts on individuals diagnosed with early-stage Parkinson’s disease (PD) through the utilization of comprehensive interviews and symptom mapping techniques. The authors identified the three most prevalent motor symptoms as tremors, fine motor difficulties, and slow movements. In contrast, the most common non-motor symptoms were nocturia, fatigue, insomnia, and cognitive changes. The work proposed in [78] employed the use of a mobile and wearable device to conveniently, non-invasively, passively, and actively quantify symptoms of PD in a user-friendly manner, allowing for the collection of data in a domestic setting. The study combined objective data collected across multiple tests with the aim of evaluating the frequency and severity of motor and non-motor symptoms. Additionally, the study sought to identify the link between data collected at home and conventional clinical scales, with the potential to enhance remote monitoring and management of PD. The objective of the project, as outlined in [79], was to develop and validate a clinical support tool for the measurement of arm swing kinematics in patients with PD. This was to be achieved through the utilization of wristbands equipped with accelerometers. The results demonstrated that the patients exhibited significantly reduced root mean square (RMS) values and elevated asymmetry in the arm swing RMS values relative to the control group. Prior research has demonstrated that individuals with PD move their arms more slowly and asymmetrically than healthy individuals. In terms of innovation, the proposed wristband prototype has taken advantage of the characteristics of portability and connectivity available in personal smartphones by establishing a communication architecture that uses them as intermediaries between the sensors and the cloud, from which the data are visible to any analysis software.

The work discussed in [80] aimed to determine whether levodopa response could be evaluated using Parkinson’s Kinetigraph (PKG) recording data with a classification performance comparable to that of the levodopa challenge test (LDCT). The authors used UPDRS III scores from before and after a levodopa dose to create an ML model that defined six categories of motor function severity. To guarantee the models’ generalizability, they are created and tested using data that has not been seen before. The obtained results suggested that PKG can be used to accurately replicate LDCT in an ambulatory fashion. In [81], the evaluation of smartwatches’ reliability and accuracy for the technology-based assessments in PD was conducted via ML classification techniques. Here, to assess tremor-like amplitudes and frequencies, a high-precision shaker, one nanometric seismometer, and two distinct series of Apple smartwatches were used to create a comparison setup. Clinical smartwatch assessments were obtained from 450 participants with PD, participants with differential diagnosis (DD), and healthy participants in prospective research. For a fifteen-minute test, each participant wore two smartwatches. Medical history and symptoms were recorded on the linked smartphone. Numerous distinct ML classifiers underwent cross-validation. Multilayer Perceptrons achieved 74.1% balanced accuracy, 86.5% precision, and 90.5% recall on the most difficult task—differentiating between PD and DD. In every categorization assignment, DL architectures fared noticeably worse than others. Also, they concluded that subtle tremor indications can be recorded by smartwatches with minimal noise. Burq et al. [82] created an active evaluation for smartwatches that allowed for the unsupervised measurement of PD motor symptoms. For a median of 390 days, participants with early-stage PD wore a commercial smartwatch and completed unsupervised motor tasks twice a week for a year, as well as once in the clinic. The incidence of dropout was 5.4%. The average wear duration was 21.1 h daily, and 59% of remote assessments were finished according to protocol. In-clinic measurements were analytically validated and showed moderate-to-strong correlates with consensus MDS-UPDRS Part III scores for gait, bradykinesia, and rest tremor. In place of clinic visits, the authors of [83] implemented remote management, including the Parkinson’s KinetiGraph (PKG™), a wrist-worn gadget that continuously measures movement. They assessed their results and reporting procedures, the type of unmet need they found, and the degree to which their treatment recommendations were followed by the patients, whose input informed the development of their services. The performed assessment brought to light both the advantages and disadvantages of integrating digital data into care that is typically provided through face-to-face interactions. Starting from the consideration that numerous studies in the literature have attempted to demonstrate the usefulness of digital measures of motor function in PD, the authors of [84] described the procedures used for evaluating the technical performance of specific accelerometers (conducted by comparing sensor output from both an iPhone and ActiGraph GT9X versus a Quanser Shake Table II) and the subsequent analytical validation of a software and algorithm combination used to compute motion measurements in healthy people. To test the algorithm’s analytical validity without considering disease, they purposefully limited the testing of human subjects to healthy people. This was an illustration of a methodical, step-by-step assessment of body-worn sensors using data processing algorithms before testing in sick patients. The project described in [85] developed a device that can be worn as a mechanical treatment for PD patients to reduce hand tremors. The human hand was modeled biodynamically by treating it as a primary mass-spring-damper system. The frequency of tremors has been matched in the design of two passive vibration absorbers. The temporal reaction of hand tremors was used to evaluate the device’s performance. According to the research results, the instrument decreased the wrist’s angular movement amplitude by 57.25%. People with PD found it simple to wear the bracelet-shaped device when necessary. Also, the work [86], which represents an evolution of the work proposed in [69], presented a novel smartwatch-based prototype, which was implemented as a prospective study in neurology. A full-stack ML pipeline using Artificial Neural Networks (ANN), Random Forests, and Support Vector Machines (SVM) was established and optimized to train for two clinically relevant classification tasks: (a) to distinguish neurodegenerative movement disorders such as PD or Essential Tremor from healthy subjects; (b) second, to distinguish specifically PD from other movement disorders or healthy subjects. Regarding the first task, ANN was the best classifier with a precision of 94%, whereas SVM performed best for the second task, with a precision of about 81%. The purpose of the study introduced in [87] was to use a widely accessible fitness tracker in a real-world setting to create an individual-level prediction model for the wearing-off of anti-PD medication. Gathering and combining the relevant datasets required for this investigation was the initial objective. Second, the wearing-off period was predicted using prediction models. Finally, to evaluate the main objective, the authors examined the results that were generated. In [88], Abrami et al. presented an unsupervised method for producing objective assessments of movement quality during at-home and in-clinic exercises. They were able to capture the growing disorder in motion between the states of health and illness by converting continuous information from wearables into a statistical distribution of movement syllables (the symbolic movement representation). Increased motor impairment in PD patients, as measured by their MDS-UPDRS scores, was likewise associated with this statistical representation. This correlation was highly accurate at predicting neurologist scores when scripted tasks were performed in clinical and in the naturalistic at-home setting. In [89], accelerometric data from a single wrist-worn device to track bradykinesia and resting tremor in PD patients was investigated. Data gathered through unscripted activities during in-clinic visits could be used by the suggested method to generate clinically useful indices of symptom severity. The amount and amplitude of hand movement significantly increase as the patient moves from the OFF to the ON state, while the consistency and amplitude of resting tremor significantly decrease. To give better interpretable data during unsupervised ambulatory monitoring, a hierarchical architecture was also used to first identify context before evaluating motor symptoms. Another interesting recent article introduced the Personalized Parkinson’s Project (PPP), a prospective single-center trial of individuals with early-stage Parkinson’s disease in the Netherlands, co-sponsored by Verily Company [90]. Participants were tracked continually through the Verily Study Watch and at 4-month intervals through on-site clinical evaluations. The authors created a PD virtual motor exam (PD-VME) with eight self-guided exercises using accelerometer data from the Study Watch. The MDS-UPDRS Part III exam, which is presently the “gold standard” for motor evaluation of PD, had components that each task relates to. Evans et al. [91] established a virtual clinic (VC) for PD patients, integrating wearable technology reports with phone consultations, to create an efficient, patient-centered, and sustainable service. The well-known wrist-worn tool Parkinson’s Kinetigraph provides objective movement assessment and generates a report that doctors use to optimize drug regimens.

The objective of the study [92] was to ascertain the feasibility and reliability of utilizing a system based on smartwatches to register rest tremor in patients with Parkinson’s disease (PD) and to evaluate its clinical correlation as a monitoring tool for PD patients in a consulting room over time. Furthermore, the authors sought to ascertain the level of acceptability associated with the system. The findings of the authors demonstrated that a Parkinsonian-related resting tremor could be quantified effectively using customized commercial hardware and exhibited a strong correlation with the resting tremor score of UPDRS-III. Furthermore, the test-retest reliability of this system was found to be good to excellent, and patients demonstrated a high level of acceptance regarding the use of a smartwatch to quantify their motor symptoms. In [93], the authors employed two datasets gathered from patients with PD, integrating continuous wrist-worn accelerometer data with regular symptom reports in a domestic setting, to develop digital biomarkers of symptom severity. The authors conducted a public benchmarking challenge, in which participants were tasked with developing severity measures for three symptoms: on/off medication, dyskinesia, and tremor. Forty-two teams participated, and performance improved over baseline models for each sub-challenge. Ensemble modeling across submissions further enhanced performance, and the top models were validated in a subset of patients whose symptoms were observed and rated by trained clinicians. Torrado et al. [94] proposed a novel user-centered paradigm of aging based on wearable-driven artificial intelligence (AI) that may facilitate the autonomy and independence that accompany functional limitation or disability, and potentially extend life expectancy in older adults and people with PD. The study included four distinct participant groups. The participants were divided into four groups: (1) individuals with PD and their informal caregivers, (2) healthy older adults from the Helgetun living environment in Norway, and (3) individuals on the Helgetun waiting list. Additionally, a comprehensive study was conducted in [95] to assess and contrast the primary technical attributes and operational capabilities of diverse commercial wrist and non-wrist devices designed to quantify tremors in patients with PD and essential tremor. In particular, the functionality of consumer devices was assessed in comparison with laboratory-grade accelerometers (LGAs). All evaluated smart devices demonstrated comparable performance to LGAs in measuring peak frequencies of tremors. However, it was not possible to make a direct comparison of the amplitude readings, as these appeared to be influenced by the specific anatomical placement of the device. In conclusion, articles pertaining to PD and wrist smart devices include the work described in [96]. The article described the “Parkinson@Home” study, a two-phase observational study involving 1000 Parkinson’s disease patients and 250 physiotherapists. The Parkinson’s Disease (PD) status of the participants was evaluated using a shortened version of the Parkinson’s Progression Markers Initiative (PPMI) protocol, which was conducted by certified physiotherapists. Furthermore, participants were equipped with a set of sensors (smartwatch, smartphone, and fall detector) and utilized these in conjunction with a bespoke smartphone application for a period of three months. The study constituted a significant stride towards the development of a dependable system that could translate and integrate real-world data.

The works discussed in this section are summarized in Table 8.

### 3.2. Epilepsy and Seizure Detection

**Figure 7:**
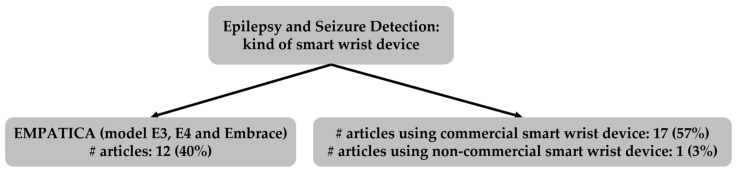
Categorization of articles related to epilepsy or seizure detection, based on type of wrist device.

Many medical studies indicate that a person with epilepsy has two or more unprovoked seizures that happen more than twenty-four hours apart. Instead, depending on which areas of the brain are affected, an excessive spike in electrical activity in the brain, known as a seizure, can produce a range of symptoms. It follows that the words “seizure disorder” and “epilepsy” are often used interchangeably. However, “provoked” seizures, such as those due to severe hypoglycemia, are not considered to be forms of epilepsy. A consequence of all the above considerations is that the articles included in the present review concerning the use of smart devices for epilepsy also discuss the use of the wrist device, commercial or otherwise, for seizure detection. In addition, analysis of the articles selected downstream of the inclusion and exclusion criteria adopted in this review showed that many researchers used the same kind of smart wrist device in their articles, manufactured by the company Empatica (https://www.empatica.com/en-eu/, accessed on 15 November 2024), that developed an automated comfortably wearable convulsive seizure detection system relying on accelerometer and electrodermal activity data. Accordingly, to facilitate the reading of the following subsection, the articles are separated into two additional subsections: (1) epilepsy and seizure detection using Empatica devices, and (2) epilepsy and seizure detection using other smart wrist devices (commercial and non-commercial). Figure 7 graphically illustrates the distribution of these articles.

#### 3.2.1. Epilepsy and Seizure Detection Using Empatica Commercial Devices

The study proposed in [97] showed that individuals with drug-resistant focal epilepsy frequently had circadian and multiday cyclical changes in a wide range of noninvasive physiologic parameters and that for most patients, the timing of seizures was associated with preferred stages of these cycles. Seizures were observed in both multiday and circadian cycles across all chronic wearable recording channels. Also, the work was particularly notable for the presence of seizure phase locking to multiday cycles in wearable recordings and the finding was like seizure phase locking to multiday cycles in brain recordings. The potential for supervised ML models to identify focal onset motor seizures in both individualized and cross-patient settings using a wearable device worn on the wrist was examined and discussed in [98]. Data were collected from epileptic patients at two epilepsy centers while they were in hospital, using wearable technology. The features for each of the biosignal modalities were calculated following the processing of pulse data from the following sources: accelerometry, electrodermal activity, and blood volume. In conclusion, the authors demonstrated that the robust detection of focal onset motor seizures with tonic or clonic movements from wearable data may be feasible for individuals, contingent on the specific seizure manifestations. The objective of the work proposed in [99] was to demonstrate the efficacy of monitoring and detecting generalized tonic-clonic seizures using a combination of accelerometers and electrodermal activity sensors. In particular, the authors described the functionality of Empatica™, a commercial smart wrist device that developed the first ML-based seizure detector on accelerometers and electrodermal activity data acquired with the E4 model. Subsequently, the technology was transferred to a stand-alone detection and alert system (Embrace). Both devices received CE medical clearance from the European Union in 2016.

Moreover, in another interesting work, accelerometry and electrodermal activity data captured by wrist-worn devices were used to create two multimodal automated convulsive seizure detectors [100]. The proposed algorithms were tested using a more varied data set than previous clinical studies, obtaining a much higher sensitivity (approximately 95%) when compared directly to the best state-of-the-art system using accelerometry and electrodermal activity. Most patients experienced less than one false alarm every four days, and 90% of patients experienced fewer false alarms than their seizure rate; no false alarms happened while they were at rest. Apart from detecting seizures, the algorithm demonstrated postictal autonomic dysfunction in 73% of cases and enabled accurate annotation of motor convulsion lengths. To improve the algorithm’s capacity to identify seizures in data from a commercial wrist-worn device, the authors of [101] presented data demonstrating that a deep neural network (DNN) LSTM algorithm can be gradually trained. This approach yielded higher accuracy than a single training cycle using wearable data alone. The progressive training method used wearable device data to train the algorithm after initial training cycles with EEG data and transfer learning. The proposed methodology demonstrated good performance on hospital-recorded tonic-clonic seizures and was able to detect hyper motor seizures with a respectable false-positive rate and reasonable sensitivity. Moreover, characterization of the LSTM algorithm on the wearable signals used suggested that accelerometer signals made the greatest contribution to the results, followed by blood volume pressure signals.

In [102], a seizure detection and pre-seizure condition detection system was developed for a wrist wearable system, namely the Empatica E4, which incorporates a photoplethysmography (PPG) sensor, an electrodermal activity sensor, an accelerometer, and temperature sensors. The seizure detection system was developed using a machine learning framework. Specifically, three supervised learning techniques, namely SVM, decision tree, and logistic regression, were compared, and the highest accuracy observed was 84.22% for SVM, 99.40% for decision tree, and 71.23% for logistic regression. One limitation of the study was the relatively small and imbalanced data set used to train the model. As the data were collected over the course of the patient’s admission, seizures occurred only for brief periods, with relatively few instances, and the seizure data set was considerably smaller than the non-seizure data set.

Using a commercial smart wrist device, the study [103] aimed to characterize extracerebral biosignal characteristics of both general and specific seizure types compared to baseline physical activities. Here, the authors created predictive models for both general and seizure types and then evaluated each model’s diagnostic efficacy. In particular, the authors verified that heart rate, acceleration, and electrodermal activity, but not temperature, were significantly elevated during seizures. Cluster analysis showed trends of the greatest elevation of heart rate and acceleration values in bilateral tonic-clonic seizures (BTCs) compared to non-BTCs and isolated auras. The pilot study reported in [104], even if in a small cohort, has shown that seizure forecasting using a noninvasive wrist-worn multimodal sensor was much better than a random predictor for most patients tested. In an ambulatory scenario, wearable data were captured while engaging in regular activities, and seizure occurrences were concurrently validated by EEG. Of the six individuals examined, five had seizure forecasts that were noticeably more accurate than a random predictor, and seizure alarms in these five patients gave enough advance notice to enhance neuromodulation therapy or give fast-acting medicine.

By a commercial wrist device, it was demonstrated in the study reported in [105] that PPG frequency showed an increase during pre- and post-seizure periods that was higher than the changes during seizure-free periods. Additionally, the PPG slope decreased during pre-seizure periods compared to seizure-free periods, and smoothness increased during the post-seizure period as compared to seizure-free periods. These results suggested to the authors that PPG analysis may offer additional information when monitoring patients with epilepsy. Using a tested seizure detection algorithm, in [106] the performance of two wearable devices based on electrocardiography and photoplethysmography is compared with a typical hospital ECG. Based on heart rate characteristics that are taken from the heart rate increase, this algorithm categorizes seizures. The sensitivity reported in the article of the wearable PPG device, the hospital system, and the wearable ECG device are 32%, 57%, and 70%, respectively, concluding that wearable ECG performance is comparable to hospital ECG performance; however, seizure detection performance with the wrist-worn PPG device was significantly lower. The authors of [107] evaluated a DL approach to predict seizures in a statistically significant manner using multimodal wristband sensor data from several epileptic patients. They found that 43% of the patients had better-than-chance prediction using a leave-one-subject-out cross-validation technique. Analyses of time-matched seizure surrogate data showed that forecasting was not solely influenced by alertness state or time of day. When all sensor modalities were employed, prediction performance was maximized. It did not differ between focal and generalized seizure types, but it did typically improve with the size of the training dataset, suggesting that future work with larger datasets may yield even greater improvements. Finally, a multicenter prospective cohort study’s main goal [108] was to evaluate the acceptability, feasibility, and usability of a wearable and mobile remote measurement technology in a community context. Furthermore, this study sought to ascertain whether multiparametric mobile remote measurement technology gathered from epileptic populations may be used to prospectively estimate differences in seizure incidence and other outcomes, such as comorbidities, quality of life, and seizure frequency. The works discussed in this section are summarized in Table 9.

#### 3.2.2. Epilepsy and Seizure Detection Using Other Smart Wrist Devices (Commercial and Non-Commercial)

In [109], the effectiveness of an Apple Watch seizure monitoring app on patients in both everyday life and an inpatient epilepsy monitoring unit setting was assessed. Patients who had a history of tonic-clonic seizures were selected for surveillance from the general outpatient population and four hospitals. A tonic-clonic seizure detection algorithm was developed and tested prospectively for 206 days. According to the results, the algorithm maintained a very low false-positive rate (0.05/day for hospitals and 0.1/day for outpatients) while operating at high sensitivity (100%), showing the efficacy of a real-time tonic-clonic seizure detection algorithm that was embedded in a consumer wearable device.

Xiong et al. [110] validated a forecasting method utilizing multimodal cycles of epileptic activity recorded on commercial smart wrist devices. In this study, seizure and heart rate cycles were extracted from the data of 13 participants, with the aim of investigating the relationship between seizure onset time and the phases of seizure and heart rate cycles. The findings of this study illustrate that cycles identified from multimodal data can be integrated into a unified, scalable seizure risk forecasting algorithm, thereby ensuring robust performance. In contrast, the authors of [111] employed a smartwatch to ascertain its potential for identifying seizure occurrences in patients in comparison to continuous EEG monitoring for those admitted to an epilepsy monitoring unit. The selected neural network models for data classification were often able to detect seizure occurrences at a level above chance, as evidenced by the patient-aggregated receiver operating characteristic curve’s area under the curve of 0.58. However, the obtained overall low specificity implied a false alarm rate that would likely render the model unsuitable in practice. In [112], the authors investigated the detection of convulsive epileptic seizures using a single accelerometer sensor worn on the wrist.

Three categories of convulsive seizures were included in the data set examined in this study: (1) psychogenic non-epileptic seizures, (2) generalized tonic-clonic seizures, and (3) complex partial seizures. The suggested system identified convulsive seizures lasting at least 10 s and only required one accelerometer sensor. Accelerometer data from patients receiving video-electroencephalography monitoring—the gold standard for epileptic seizure identification—was used to validate the suggested algorithm. To train Kernelized support vector data description, a new set of computationally efficient time-domain features—including features extracted using a non-linear method—was utilized to classify seizure and non-seizure events, detecting roughly 87% of the three types of seizures.

A recently conducted study [113] employed a mixed methods design to assess the direct experiences of individuals with epilepsy who used a non-invasive monitoring system, EEG@HOME, for an extended period of six months in their own homes. The study had three principal objectives: to investigate factors affecting engagement, to gather qualitative insights, and to provide recommendations for future home epilepsy monitoring systems. The results demonstrated the enthusiasm and aptitude of individuals with epilepsy for active health monitoring with new technology. It was concluded that independent home use of new non-invasive technologies can be made possible by remote training and assistance. However, to ensure long-term acceptability and usability, systems must be incorporated into patients’ daily routines, include healthcare providers, and provide ongoing support and tailored feedback.

The authors of [114] designed and developed an electronic device and data collection system for epilepsy and seizure detection, and they investigated and proved the practicality of the newly proposed device and methodology for data classification. Using the proposed smart bracelet, they gathered information from epileptics outside of the hospital. Following a seizure, the individuals were instructed to hit the mark button. To eliminate nonmoving segments, the authors also introduced an automated extraction and annotation of the moving segments technique. Next, they classified seizure and non-seizure movement segments using a two-layer ensemble model and ML techniques, achieving about 77% sensitivity and 97% accuracy in data classification. The main objective of the work proposed in [115] was to examine the features of motor manifestation during psychogenic nonepileptic seizures and convulsive epileptic seizures, as recorded by a wrist-worn accelerometer device. Finding quantifiable accelerometer characteristics that can distinguish between convulsive epilepsy and convulsive psychogenic nonepileptic seizures was the primary objective. Two new indices—tonic index and dispersion decay index—were used to quantify the Poincaré-derived temporal variations for every generalized tonic-clonic seizure and convulsive psychogenic nonepileptic seizure event. The authors concluded that an automated classifier built using the features differentiated convulsive psychogenic nonepileptic seizure events with a sensitivity of about 95.5% and classified generalized tonic-clonic seizures with a specificity of 95%. Ge et al. [116] showed in another very interesting work how mobile devices might be used to track seizures and complete postictal surveys to find seizure triggers in a heterogeneous, nationwide population with epilepsy. 26% of all seizures were linked to different triggers, and 41% of participants who tracked seizures reported seizure triggers. According to people with epilepsy in this study, stress was the most frequent cause of their seizures, followed by sleep deprivation and correlations with the menstrual cycle. However, many participants with seizure triggers noted that a combination of circumstances, most frequently stress and other factors like fatigue or lack of sleep, can cause seizures. This implied that these variables used together may change seizure thresholds and affect seizure timing and risk. The authors of [117] tested a wrist-worn smart device on children, adolescents, and young adults with various types of seizures in an epilepsy monitoring unit. Confirmation of seizure type and if there was rhythmic upper extremity jerking associated with the seizure was determined by a review of the video EEG. This was compared with the standard detection system of the considered commercial smartwatch, which detected only 16% of the total seizures, 31% of the generalized tonic-clonic seizures, and 34% of seizures associated with rhythmic arm movements. In [118], a pilot study on the impact of quality of life for adolescents with epilepsy and their caregivers was described. Throughout the study period, there was a trend toward improvement in the overall quality of life measures of adolescents, as well as greater support for parental autonomy. According to the findings, adolescents with epilepsy and their caregivers were open to utilizing the commercial seizure detection device, despite certain restrictions with the SmartWatch. Moreover, according to the study’s findings, seizure detection devices can help to live better reducing worry related to seizure safety and normalizing the natural developmental process of adolescents becoming independent from their families. Lazeron et al. [119] validated, for children living at home and in residential care, a wearable seizure detection device, based on PPG and accelerometer data for detection of nocturnal major motor seizures. The smart device was previously tested only on adults. From the conclusion it has emerged that the sensitivity of the device was comparable in adults and children, and in general, children exhibited an increased number of false alarms, which were typically triggered while they were awake, but the authors concluded that the number of false alerts could be reduced to a level that was comparable to that of adults by adjusting the algorithm’s sensitivity for body position. In [120], the utility of electronic self-reported seizure diaries and non-invasive wearable physiological sensor data to estimate seizure risk in retrospective and pseudo-prospective cohorts was assessed. The results reported in the article have demonstrated that non-invasive wearable sensors in the field of seizure forecasting were not only possible but feasible and imminent. An evident limitation was that self-reported seizure diaries had inherent drawbacks and were known to be inaccurate. Not everyone with epilepsy was aware of when they experienced a seizure, particularly if they predominantly experienced focal-aware seizures. Also, a wrist-worn device was used to collect accelerometer data from patients in [121] for diagnostic evaluation of convulsive seizures. Specifically, K-means clustering and SVM were employed in an automated procedure to identify and categorize each seizure as either epileptic seizures or psychogenic non-epileptic seizures. Epileptologists who were blinded to the accelerometer data compared the results with video EEG monitoring diagnoses. The results reported a sensitivity and specificity value for classifying epileptic seizures from psychogenic non-epileptic seizures of about 72.7% and 100%, respectively, whereas the positive and negative predictive values for classifying psychogenic non-epileptic seizures were 81.3% and 100%, respectively. A multicenter, in-home, prospective, video-controlled cohort study was proposed in [122], wherein people who had epilepsy intellectual disability, and nocturnal seizures were identified by movement (3-D accelerometry) or heart rate (PPG). Approximately 82% of the initial study participants completed the trial with the following results: median sensitivity per participant amounted to 86%, the false-negative alarm rate was 0.03 per night, and the positive predictive value was 49%, concluding that the combination of heart rate and movement resulted in reliable detection of a broad range of nocturnal seizures. Van de Vel et al. [123] evaluated four different systems (including a smart mattress and a smart wrist device) based on efficiency, comfort, and user-friendliness and compared them to one patient suffering from focal epilepsy with secondary generalization. Even though nongeneralized and nonrhythmic motor seizures (involving only the head, having a tonic phase, or presenting primarily as sound) were frequently ignored, some of the devices had good results. In addition to its ease of use (few setup steps), comfort (contactless), and ability to customize patient-specific settings, the smart mattress was selected for the only selected patient for the experimentation stage. A novel framework was proposed in [124]. It used an IoT and Blockchain oversight mechanism to augment the Vagus Nerve Stimulator (VNS), a tool for treating epileptic episodes that sends counter-electrical stimulations to the Vagus Nerve. The deployed system was able to make decisions and regulate the use of the VNS. A smartphone application that enabled patients and caregivers to safely report seizures in real-time to the appropriate epilepsy care team has been evaluated in [125], where the capacity of wrist-worn devices to identify epileptic episodes was also assessed. The epilepsy care team securely received pertinent information in real time, including seizure notifications and live alerts for emergency attendance or hospital admission. The time between a seizure occurring in the community and the expert team being notified decreased and guidance was given more quickly than in the year before the pilot. Patients with epilepsy experienced a 10% decrease in length of stay and a 30% decrease in admissions. Patients who use the technology say they feel more empowered. This treatment approach has several drawbacks and necessitates changing current procedures if patient advantages are to be fully realized. The performance of an accelerometry-based wearable system to detect tonic-clonic seizures was evaluated by Johansson et al. [126]; also, the authors investigated the accuracy of different seizure detection algorithms using separate training and test data sets. The training data was used to construct and train algorithms that combined a tonic-clonic seizure event detection layer with regular binary classifiers. Sensitivity and false positives were used to assess their performance, concluding that the highest sensitivity was obtained for the k-Nearest Neighbor, and the lowest false-positive rate was obtained for the RF binary classifier.

The works discussed in this section are summarized in Table 10.

### 3.3. Essential Tremor

Essential tremor is one of the most common movement disorders typified by uncontrollable, rhythmic shaking of body components. A thorough and precise evaluation of tremor severity is necessary for essential tremor to be diagnosed and treated properly. Conventional approaches rely on rating scales and clinical observation, which might create subjective biases and impede ongoing assessment of illness development. Recent studies have investigated new methods for measuring essential tremor. Using intelligent smart devices to enable quantitative and objective measurements is a potential approach.

For example, in [127], tremors were regularly monitored during daily life using brand-new, highly portable technological devices. It is made up of a smartphone, a remote server, and a smartwatch with a triaxial accelerometer.

The study was conducted with eight patients. A total of eight patients were included in the investigation. Significant correlations were observed between the neurologist’s Fahn–Tolosa–Marin Tremor Rating Scale (which was adopted as the gold standard) and the device’s quantitative measures. This demonstrates the feasibility of prolonged and continuous monitoring of tremor severity during daily activities by a highly portable non-restrictive system, which could prove to be a useful tool for the analysis of the efficacy and effectiveness of treatment. The objective of the study proposed in [128] was to differentiate essential tremor from Parkinson’s disease using a smartwatch device in an outpatient clinic setting. Recordings were obtained using a smartwatch device on the hand predominantly affected by the tremor (in all patients), and simultaneously with an analogue accelerometer (in 10 patients) with the hands at rest and outstretched. The tremor peak frequency, peak power, and power of the first four harmonics were calculated and compared between the two devices. The mean power at the first four harmonics was calculated and used to classify tremors as Parkinsonian or essential tremors. The results indicated that the mean harmonic peak power was both highly sensitive and specific for distinguishing PD tremor from essential tremor, with an optimal threshold for the considered sample. In a pioneering randomized pragmatic clinical trial, Dai et al. [129] compared Transcutaneous Afferent Patterned Stimulation (TAPS) with the standard of care (SOC) in patients with essential tremor. The study demonstrated that the addition of TAPS therapy to SOC resulted in a significant improvement in tremor power compared to SOC alone during a one-month period of home use. Furthermore, the findings highlight the potential of motion sensors in measuring tremor power over extended periods of home use, and as an endpoint for evaluating the effectiveness of TAPS therapy or other interventions for essential tremor in clinical trials. In a recent study, the authors of [130] evaluated the efficacy and safety of a peripheral nerve stimulation device worn on the wrist in patients with essential tremor. The tremor was assessed before and immediately after the conclusion of a single 40-minute stimulation session. The findings indicated that peripheral nerve stimulation may provide a safe, well-tolerated, and effective treatment for the transient relief of hand tremor symptoms in patients with essential tremor.

Using motion sensors and deep learning algorithms, an automated reference scale system was developed in [131] to precisely rate the intensity of essential tremor during voluntary activities. Twenty participants’ motion data was gathered as they performed everyday tasks using a tremor monitoring device that was based on a smartwatch. To differentiate between voluntary human actions and assess the intensity of tremors, respectively, activity classification models and tremor evaluation models were developed, and algorithms were designed and implemented with the use of DL techniques. This study showed how motion sensor data may be utilized to categorize human activities and assess the degree of essential tremor during various tasks. The goal of [132] was to find out how various accelerometry measurements may be used to objectively categorize tremors in PD and tremor amplitude in essential tremor. Under various deep brain stimulation conditions, the authors of the work evaluated 860 resting and postural tremor trials in a total of 41 patients (16 essential tremor patients and 25 PD patients), concluding that triaxial accelerometry reliably quantified resting and postural tremor amplitude in both categories of patients. The method described in [133] detected tremors by using accelerometer data collected in the field. A set of acceleration signal segments and a unique tremor label were used to represent everyone. The thorough testing of a patient and non-patient dataset showed that the implemented method could detect these occurrences. Furthermore, the approach might effectively tackle the problem of insufficient supervision because it was trained solely with the existing insufficient subject-level annotations. Lastly, it outperformed the alternatives that were taken into consideration. By comparing regular neurological evaluation with a “Tremor Occurrence Score” obtained from smartwatch sensor data, van Alen et al. [134] were able to resolve the limitations of subjective clinical tremor assessment in 142 patients with PD and 77 healthy controls. Reported results demonstrated the potential of smartwatches for automated tremor detection as a useful supplement to traditional evaluations, suitable for use in both home and clinical contexts.

Also, the authors of [135] measured objective tremor duration and subjective symptom burden in patients with functional tremor or organic tremor using a smart wrist device, for one month in their home environment. In individuals with functional tremor, the authors found a significant amount of objective tremor duration. Mainly patients with essential tremor, patients with organic tremor had a statistically significant greater degree of objective tremor duration (30.7% of the time). Additionally, there was no statistically significant difference in the subjective symptom load between the two categories of patients considered in the study. Lastly, no difference in the relationship between subjective and objective symptoms between the functional and organic tremor groups was found. The prospective study reported in [136] was one of the largest clinical trials in essential tremor to date, with 263 patients enrolled across 26 sites (but only 205 of the 263 enrolled patients completed their third in-clinic visit and were included in the primary endpoint analysis). According to this study, many patients with essential tremor may safely and effectively reduce their hand tremors by using non-invasive neuromodulation therapy on a regular basis over a period of three months at home. López-Blanco et al. [137] presented a clinical investigation to assess the viability, clinical correlation, and dependability of quantifying tremors in patients with essential tremor using a smartwatch-based system and determining whether it is accepted as a clinical monitoring tool. Clinical tremor scores and smartwatch assessments of tremor severity had Spearman’s correlation coefficients of 0.590 at rest and 0.738 in steady posture; also, smartwatch reliability was checked by intraclass reliability coefficients: 0.85, and 0.95, respectively. Moreover, a retrospective post-market surveillance study evaluated the real-world effectiveness of TAPS, a wrist-worn device-delivered, non-invasive neuromodulation therapy for the treatment of hand tremor in patients with essential tremor [138]. Over the course of all experiments, TAPS decreased tremor power by 71%, and 59% of patients reported a reduction in tremor of at least 50%. Notably, 65% of patients who completed the voluntary survey said their quality of life had improved, and 84% said they had improved at least one of their eating, drinking, or writing skills. The last article in this subsection presented a passive absorber device for attenuating pronation/supination tremor [139]. It was based on the principles of dynamic vibration absorption and is tuned to the frequency of the tremor. Prototypes were manufactured and tested on a mechanical model of the human forearm. Simulations and experiments demonstrate the efficiency of the device in attenuating vibrations in the range of 4–6 Hz, which is the range of frequency of observed tremors, with a maximum amplitude attenuation of 85%.

The works discussed in this section are summarized in Table 11.

### 3.4. CP/UCP

From a scientific research perspective, CP is an “umbrella term”, meaning that it is a name given to a group of disorders and functions. CP affects different parts of the body. (1) Bilateral CP involves both sides of the body and can also be described as Diplegia (where the legs are more affected than the arms) and Quadriplegia (where the whole body is involved); (2) UCP or Hemiplegia means one side of the body is affected—either the left side or the right side. Most of the scientific papers considered in this review fall into the latter category of CP.

The accuracy of measuring the unilateral hand’s functional usage in children with CP using a dual wrist-worn accelerometry and ML approach is reported in the study [140]. The random forest (RF) classifier was preferred because of its significantly better performance in the designed validation scenario. While more research is required to increase the generalizability of the adopted strategy before it is widely adopted, the work indicated that the ML approach may perform better than the activity count approach in predicting the functional use of the unilateral hand in children with CP. According to [141], people with UCP and their families tolerated the use of a commercial wrist-worn device and a smartphone app designed to encourage more use of the afflicted arm in daily life. From the results obtained, it has emerged that the method did not result in a sustained increase in activity of the damaged limb, and changes were necessary to address technological concerns. For long-term benefits, a therapy program and more supervision of the strategy would probably be required. Some participants found the buddy system to be motivating, and this should be investigated further in subsequent research.

To identify asymmetries in bilateral real-world arm activity at baseline and following intensive occupational therapy interventions to improve arm function, children with UCP can benefit from an accurate, user-friendly, and objective assessment tool that wrist-worn accelerometry offers. This is demonstrated by the pilot study reported in [142]. In this study, the authors compared the intensity and duration of activity in the dominant or unaffected upper extremity (UE) with that in the non-dominant or affected UE, using wrist-worn accelerometry for a seven-day period with nine typically developing (TD) children and nine children with UCP. Those with UCP demonstrated a reduction in relative activity within the non-dominant UE in comparison to the dominant UE, in contrast to the findings observed in typically developing (TD) children. The use of accelerometers on the wrist proved to be an effective method for identifying asymmetries in bilateral all-day UE use in children. The overarching objective of the project delineated in [143] was to evaluate the efficacy of a single joystick-operated ride-on toy navigation training (RNT) program in facilitating the enhancement of affected UE function in young children with UCP. A combination of standardized, quantitative, and caregiver-report measures was employed to assess changes in manual abilities and upper extremity function. The article did not present the results of the study, but rather the expected outcomes. Despite the authors identifying preliminary limitations, including the lack of a separate control group, the small sample size, and the challenges in generalizing the findings to children with UCP with varied motor presentations, the expected outcomes were presented. The objective of [144] was twofold: firstly, to ascertain whether accelerometry characteristics align with hand function as measured by the Hand Assessment in Infants (HAI) tool, developed in 2017; and secondly, to investigate the feasibility of utilizing two AX3 Axivity monitors in wrist-worn bands to quantify movements in newborns at high risk of unilateral CP.

In terms of practical use, it was discovered that it was relatively possible to measure the motions of both upper limbs in infants with unilateral brain injury by using specially designed soft bracelets with accelerometers. It was challenging to give these metrics a trustworthy meaning, nevertheless, because the study of the accelerometry data revealed a considerable degree of fluctuation in the actimetry parameters. The use of actimetry in real-life upper limb movements to identify clinically significant dysfunction or asymmetries or to track their development in infants under one year of age was generally not supported by the limited correlations found between actimetry measured during spontaneous activity at home and upper limb function and potential asymmetry measured by the HAI. Gardas et al. [145] in their article use accelerometers to measure the characteristics of bimanual performance (activities and participation) in real-world contexts and bimanual movement intensity after 30 h of hand-arm bimanual intensive therapy (HABIT) in children with UCP. Six common accelerometer-derived variables were used to quantify movement intensity and performance improvements. Standardized hand function tests were used to evaluate bimanual capacity (body function and activities). During HABIT, the authors observed a significant rise in accelerometer variables, which suggested increased bimanual symmetry and intensity. Children showed notable advances in all accelerometer parameters after HABIT, which reflected improvements in real-world performance. After HABIT, children also experienced notable and clinically meaningful improvements in hand capacity. Consequently, the results implied that accelerometers could measure the intensity of bimanual movement during HABIT objectively.

The work discussed in [146] aimed to explore the feasibility of using time-matched uniaxial accelerometers for measuring movement in daily life in children with CP before and after botulinum toxin injections. All the accelerometers were time-matched to determine bimanual activity, voluntary arm movement, arm swing, and ambulation. With this configuration, the viability of wearing accelerometers was assessed. The percentage of time and the intensity at which the various activities were completed were analyzed using a linear mixed model. Results highlighted that time-matching of accelerometers placed on both wrists, the waist, and one ankle is a feasible method of registering ambulation, arm swing during gait, and arm movements while not ambulating. On the other hand, the article’s findings reported in [147] emphasized how crucial it is to carefully select the metric, activity count threshold, and epoch duration combination when processing accelerometry data for upper extremity quantification in subjects with CP. It was also crucial to take these parameters into account when comparing research in the literature. Compared to the epoch length of 2, smaller activity count thresholds were needed for epoch lengths of 1 and 1.5. The findings also implied that when choosing a suitable thresholding technique, the participant’s height should be considered. The results indicated that a lower criterion should be applied when evaluating youngsters. All things considered, the paper questioned the notion that upper extremity relative use can be measured using extremely low thresholds, such as the one frequently employed in the literature. The last article reviewed and belonging to this subsection of the review determined the efficacy of web-based training on activity capacity and performance in children with UCP. Independently, ambulant children and adolescents with UCP were randomized to receive either routine care (waitlist control) or 30 min of training (intervention) six days a week for 20 weeks in a matched-pairs randomized waitlist-controlled study. The 6-minute walk test and maximal repetitions of functional strength tests were used to evaluate activity capacity. Data were analyzed to compare groups using hierarchical linear modeling. From the reported findings, it was determined that training was effective at increasing functional strength and walking endurance in independently ambulant children with UCP, and this did not translate into improvements in activity performance.

The works discussed in this section are summarized in Table 12.

### 3.5. Other Movement Disorders (Huntington’s Disease, Gait Disorders, Tourette Syndrome, Ataxia)

Some movement disorders have been treated sporadically in the past decade. In fact, for some disorders, the present review work identified several publications less than or equal to four.

Relative to HD, four publications were identified, briefly described below. In [149], an evaluation of the potential to objectively quantify chorea in HD patients using wearable sensor data was provided. Specifically, the study proposed by the authors was designed to provide high-resolution data to inform the design of predictive algorithms for chorea quantification, demonstrating that arm chorea can be characterized using accelerometer data during static assessments. However, the model’s generalizability was constrained by the limited sample size. The degree of chorea was also highly correlated with the severity of chorea as reported by patients and doctors, according to the sensor-based model. Furthermore, the investigation revealed that each patient had a unique chorea digital signature. Lipsmeier et al. [150] created a remote digital monitoring platform based on smartphones and smartwatches to evaluate the behavioral, cognitive, motor, and functional domains in HD through frequent active and continuous passive monitoring. The feasibility, reliability, and cross-sectional validity of this platform for tracking motor and cognitive features (two important domains that alter with clinical progression across adult HD) were assessed in individuals with premanifest HD (those genetically confirmed to have HD but not exhibiting diagnostic motor symptoms of HD), manifest HD (those with diagnostic motor symptoms of HD), and control participants. Standard in-clinic evaluations showed a strong correlation between the chosen features and both clinical severity and disease stage. Across three selected studies, overall adherence to the active tests ranged from satisfactory to exceptional, indicating that most participants found the duration and quantity of daily tests to be acceptable. The study proposed in [151] presented measures of daily walking in individuals with evident HD that were determined by an accelerometer. In a novel approach, the authors evaluated everyday walking in the community among individuals with HD while taking into consideration involuntary, irregular movements using wrist-worn devices. When employing accelerometer-derived data for remote monitoring in HD, this study considered the possible impact of involuntary movements during walking. Among a diverse sample of HD subjects, it was discovered that daily living step counts and walking duration were not different from those of their contemporaries without HD; however, these metrics were inversely connected with the severity of the disease.

Four articles also describe the correlation between gait disorders and smart wrist devices. In [152], an authentication method based on gait analysis at the wrist has been presented and evaluated. The method relied on the acceleration signal and used anomaly detection to understand if the current user is the legitimate one (i.e., the owner of a wearable device) or someone else. The experimental evaluation showed that the method is reliable, and that authentication can be executed with an equal error rate (EER) of 2.5%. Also, through the analysis of inertial measuring unit (IMU) data, the study reported in [153] proposed a non-invasive technique for identifying Freezing of Gait (FoG) episodes. Window-based detection of the FoG events was achieved by processing accelerometer and gyroscope data from 11 PD individuals, which were obtained from a single wrist-worn IMU sensor while they were walking continuously, using a DL approach. The effectiveness of the suggested method, DeepFoG, in accurately estimating the presence or absence of a FoG episode at each data window was assessed using 10-fold cross-validation (CV) and Leave-One-Subject-Out (LOSO) CV methods. According to experimental results, DeepFoG operated satisfactorily, achieving 83%/88% sensitivity/specificity for LOSO CV and 86%/90% sensitivity/specificity for 10-fold CV schemes. Kiprijanovska et al. [154] proposed a new approach to identify anomalies in gait using a deep neural network and a wrist-worn device. It successfully learned spatiotemporal properties from numerous sensor signals by combining convolutional and bidirectional long short-term memory (LSTM) layers. Data from 18 participants who wore impairment glasses and recorded both their normal and aberrant gaits were used to assess the suggested approach. IMU sensor signals from smartwatches the subjects wore on both wrists make up the data. Using data from an accelerometer, gyroscope, and rotation vector sensor, the suggested method detected irregular walking patterns with 88.9% accuracy, 90.6% sensitivity, and 86.2% specificity, outperforming the comparative methods in numerous studies. To identify deterioration in gait, the main objective of [155] was to compare the accelerometer placement on wristbands with that of a cell phone in a pouch. All methods depended on a single 3D accelerometer and employed the same standard ML algorithms. The authors chose the two wristbands based on their knowledge of how they work and to clarify any discrepancies in the wristband technique. It is important to mention that other researchers can utilize the newly acquired dataset presented in the work, which included 17 test individuals, for benchmarking.

The last two articles included in this review are about two movement disorders treated exclusively in one article. Lipsmeier et al. [156] investigated the idea that movement patterns taken from continuous wrist accelerometer data capture ataxia-telangiectasia disease progression and motor impairment. A novel algorithm was developed to extract wrist sub-movements from the velocity time series. The findings supported the potential of passive wrist sensors as ecologically valid motor biomarkers by demonstrating that the data were interpretable, dependable, and sensitive to changes in illness. Regardless of location or socioeconomic background, the ability to obtain these measurements via a cheap sensor found in many smartwatches may help to promote neurological care and research participation. Finally, the last article [157] aimed to evaluate the efficacy of a home-administered neuromodulation treatment for tics involving the delivery of rhythmic pulse trains of median nerve stimulation (MNS) via a wearable ‘watch-like’ device worn at the wrist. The authors examined tics since their occurrences characterized two neurological disorders: Tourette syndrome and chronic tic disorder. The results demonstrated that after 4weeks of stimulation, tic severity had reduced by 7.1%. These findings indicated that home-administered rhythmic MNS delivered through a wearable wrist-worn device has the potential to be an effective community-based treatment for tic disorders.

The works discussed in this section are summarized in Table 13.

### 3.6. Raw Data Extracted and Classification Methodologies

**Figure 8:**
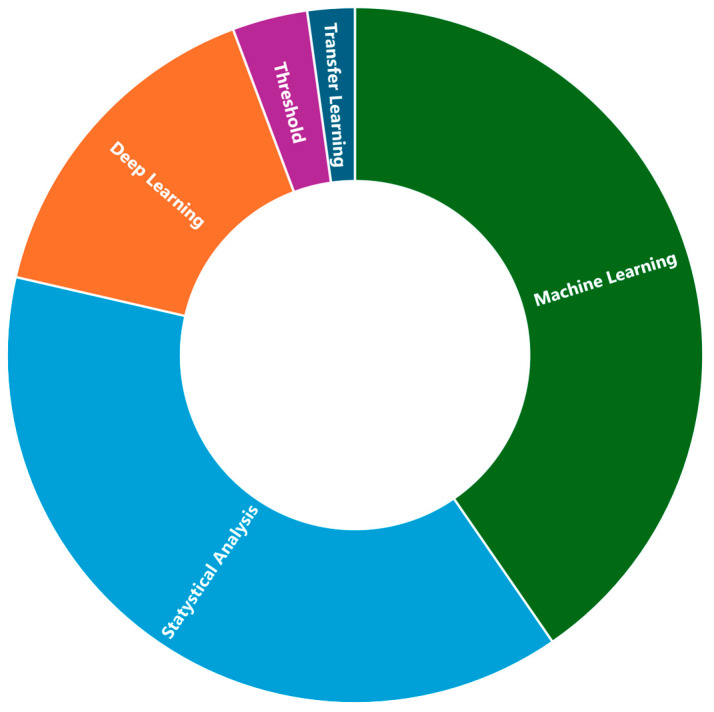
Graphical representation of the distribution of articles with respect to classification methodologies.

Another very important aspect that has emerged as a result of the analysis of the scientific articles included in this review relates to the use of the raw data provided by the smart wrist devices, the latter being useful for the next stage of classification of movement disorders. In general, the most used raw signal is undoubtedly the accelerometric one (present in about 87% of the analyzed publications), followed at a distance by the data extracted from the gyroscopic sensor (present in 16% of the articles). Finally, the analysis regarding the use of raw signals showed that electrodermal activity (EDA) and photoplethysmography signal (PPG) are used most for the evaluation of epilepsy or seizure detection. Moreover, with respect to the topic considered in this review article, it was found that most of the articles report classification results of data acquired through wrist smart devices. Among the classification methodologies, some articles use threshold approaches (3.5%) while a larger number of articles (especially the more recent ones) use artificial intelligence methodologies based on Machine Learning (40.4%), Deep Learning (15.7%), and Transfer Learning (2.2%). The review also includes a good number of articles in which data classification is conducted based on statistical analysis performed either on the raw data or on a subset of features extracted from the same data (38.2%). All the data reported above are schematized by the graph shown in Figure 8.

## 4. Other Reviews on Movement Disorders or Use of Smart Wrist Devices in Different Contexts

This section briefly discusses some recent reviews relevant to the topics covered in this article. For instance, in [158], recent trends in commercial technologies for monitoring and managing risks in the work environment are examined. Workplace health devices were analyzed considering work-related musculoskeletal disorders, functional movement disorders, cardiovascular health, respiratory risks, and continuous glucose monitoring, highlighting the benefits of real-time analysis of workers. In [159], the use of wrist-wearable devices in sports is considered to understand their potential and identify new challenges and future lines of research related to this technology. The work is focused on the use of wrist-wearable technology (a) for comprehensive monitoring of athletes’ behavior in non-provider-supported activities, (b) to detect specific types of movements or actions in specific sports, and (c) to predict injuries. The authors conclude that a promising research direction could be to evolve from low-/mid-level processes and services (such as raw data monitoring or performance and behavior analysis) to high-level services like recommendation mechanisms. The review described in [160] is concentrated on the diagnostic uses of wearables worn on the wrist to identify a variety of diseases, including metabolic disorders like diabetes, neurological disorders, fatty liver diseases, cardiovascular diseases, sleep disorders, and psychological disorders. The authors affirmed that effective wearable use necessitates quick and perceptive data processing, which machine learning makes possible. Consequently, they also covered several machine-learning applications and results for wearable data analysis in the proposed review. Finally, the authors discussed the current challenges with wearable usage and data, and the future perspectives of wearable devices as diagnostic tools for research and personalized healthcare domains. Other reviews instead considered the application of wearable sensors on specific disorders as in [161,162]. Specifically, [161] focuses on the use of wrist devices in the management of Parkinson’s disease. The authors demonstrated that much research has been performed showing promising results to involve wearable devices to achieve an accurate wellness view of PD patients. Finally, they suggest the inclusion of emerging technologies such as nanotechnology and embedded sensors which may improve the usability of wearable devices to provide more accurate results [162] focuses on the clinical utility of wearable devices in the diagnosis and monitoring of neurological and psychiatric disorders such as autism spectrum disorder, Parkinson’s disease, etc. In addition, to increase the value of wearable devices in the monitoring and diagnosis of these diseases, an ever-growing collaboration between patients, physicians, researchers, and technical personnel is suggested. From the analysis of the reported reviews, it can be seen that previous work has been limited to certain pathologies (e.g., Parkinson’s disease) or to no healthcare domains (e.g., work or sport) or has used wearable sensors that are not necessarily wrist worn. In contrast, this review focuses on analyzing the use of wrist-worn devices in the detection/monitoring of many pathologies, also considering their user-friendliness for elderly or frail individuals.

## 5. Challenges and Open Research Issues

The pervasive integration of smart wrist devices into the global population has created a unique opportunity for individuals, researchers, and clinicians to leverage the vast repository of physiological and activity data collected by these devices in naturalistic settings to monitor movement disorders. The increasing use of smartwatches and bracelets for health monitoring purposes (beyond the detection of movement disorders) has led to a growing need to assess their measurement precision against gold-standard references. Currently, the use of consumer smartwatches in health applications is limited by the unknown data quality of the sensors integrated into the same, which is the reason why only a small number of wrist devices on the market have been medically certified.

A careful analysis of the articles presented in the review also shows that there are still difficulties in using smart wrist devices, both commercial and non-commercial, for clinical research and treatment of movement disorders, despite their many benefits and prospects.

In fact, today’s wrist devices are mostly used for fitness and wellness applications by most of the population. Doctors still do not use or “trust” the data produced by a commercial smart wrist device. How it is possible to improve the accuracy and precision of data useful for the detection of a movement disorder would consequently be a new challenge to solve.

In many papers reviewed, it was found that the acceptability of a movement disorder monitoring solution through smart wrist devices is superior to that of other wearable devices, keeping in mind, however, that often a problem for frail and/or elderly users is that they forget to wear the device, in some cases not providing necessary continuous monitoring of certain indicative parameters to assess changes concerning a specific movement disorder. Another important consideration that emerged is related to the high percentage of research papers in which the wrist device used is non-commercial (approximately 20%). The reflection that emerges from this data is that many commercial devices probably do not provide direct access to the raw data, some providing it exclusively by paying monthly or annual subscriptions to proprietary platforms, in some cases preventing widespread deployment of a motion disturbance monitoring solution.

Another challenge that has emerged from analyzing scientific publications is related to the need in most cases to own not only a wrist device but also a smartphone with the functions of acquiring the raw data, processing it, and transmitting it to a cloud platform. Solutions that take advantage of this technological configuration could present the following issues: (1) lack of connection, (2) high distance between the smartphone and smartwatch resulting in the loss of data packets, and (3) battery life of the device.

For medically recognized movement disorders, the review found that only a small number of these have been investigated using smart wrist devices. Most of the papers (about 50%) concern PD, certainly one of the most prevalent movement disorders in the world population. It is evident, however, that disorders such as HD, ataxia, and Tourette Syndrome have received little attention from the scientific community studying the use of wearable device data (not only wrist devices) to detect and monitor such movement disorders.

Considering the nature and significance of this study, it is essential to address any constraints that might affect how the results are interpreted and applied generally. First, the overall validity of the selected study may be affected by the lack of a specific quality assessment. The absence of a comprehensive evaluation of the included articles’ quality adds variability that needs to be considered, even when the PRISMA-S technique is used for the systematic review. The limitations that result from this omission should be acknowledged by the authors, who should also be aware that the strength of the results made may be impacted by the varying quality of the evaluated study.

Furthermore, the limited coverage of datasets included in this review is a significant constraint. Despite helping cover a large volume of literature, the focus on Scopus, PubMed, and IEEE Explorer may be biased because it could leave out pertinent research that is probably already available in other databases. Because of this constraint, the review’s comprehensiveness and representativeness may be compromised since pertinent research may have been inadvertently left out. It is important to acknowledge this limitation, and the authors need to suggest that future studies broaden the search to encompass other databases.

## 6. Conclusions

This comprehensive review has meticulously examined the use of smart wrist devices for the assessment of a specific class of movement disorders, delving into its various dimensions and identifying both the challenges and opportunities that lie ahead for future research. Through a careful selection process, scientific publications relevant to the topic were analyzed, excluding many works considered inconsistent or with non-quality scientific content. It is believed that this review work is fundamental to understanding the current panorama of the use of commercial and non-commercial wrist wearable devices for the evaluation of specific movement disorders. An accurate analysis of the publication dates of the articles also demonstrates how there is a growing interest in the topic investigated, with analyzed works no older than 10 years, and with almost exponential growth in the last 5 years mainly motivated by a greater need for remote monitoring to encourage faster diffusion of the implemented solutions.

The work conducted also aimed to identify the challenges that need to be addressed and to highlight potential paths for future research and innovation. This review, therefore, contributes significantly to the ongoing academic debate in the field and constitutes a valuable resource for researchers, developers, and practitioners.

Overall, we have included an important number of publications in the present review, but many of these have been validated in controlled contexts, so they need further development and evaluation prior to implementation in clinical practice. The field gravitates towards the evaluation of PD through smart wrist devices, and a narrow class of other movement disorders, leading to widespread disorders within the world population. We encourage collaboration within the field and the reuse and improvement of already existing technological solutions, to prevent reinventions of the wheel and premature termination of development efforts.

## Figures and Tables

**Table 1:** Search strategy at varying of each considered multidisciplinary database.

Database	Query
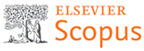	TITLE-ABS (((“Smartwatch” OR “Smartwatches” OR “Wristband” OR “Wristbands” OR “Bracelet” OR “Bracelets” OR “Smart watch” OR “Wrist-worn” OR “Wrist device” OR “Wrist devices” OR “Actigraph” OR “Apple watch” OR “Garmin” OR “Fitbit”) AND (“Movement disorder” OR “Movement disorders” OR “Gait disorder” OR “Gait disorders” OR “Gait” OR “Parkinson’s disease” OR “Parkinson” OR “Parkinson’s” OR “Movement disease” OR “Ataxia” OR “Dystonia” OR “Essential Tremor” OR “Huntington’s Disease” OR “Multiple System Atrophy” OR “MSA” OR “Myoclonus” OR “Progressive Supranuclear Palsy” OR “PSP” OR “Unilateral cerebral Palsy” OR “UCP” OR “Rett Syndrome” OR “Secondary Parkinsonism” OR “Spasticity” OR “Tardive Dyskinesia” OR “TD” OR “Tourette Syndrome” OR “Tics” OR “Wilson’s Disease” OR “Chorea” OR “Epilepsy” OR “Seizure”))) (LIMIT-TO (DOCTYPE, “ar”))
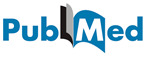	((Smartwatch [Title/Abstract]) OR (Smartwatches [Title/Abstract]) OR (Wristband [Title/Abstract]) OR (Wristbands [Title/Abstract]) OR (Bracelet [Title/Abstract]) OR (Bracelets [Title/Abstract]) OR (Smart watch [Title/Abstract]) OR (Wrist-worn [Title/Abstract]) OR (Wrist device [Title/Abstract]) OR (Wrist devices [Title/Abstract]) OR (Actigraph [Title/Abstract]) OR (Apple watch [Title/Abstract]) OR (Garmin [Title/Abstract]) OR (Fitbit [Title/Abstract])) AND ((Movement Disorder [Title/Abstract]) OR ((Movement Disorders [Title/Abstract]) OR (Gait disorder [Title/Abstract]) OR (Gait disorders [Title/Abstract]) OR (Gait [Title/Abstract]) OR (Movement disease [Title/Abstract]) OR (Parkinson’s disease [Title/Abstract]) OR (Parkinson [Title/Abstract]) OR (Parkinson’s [Title/Abstract]) OR (Ataxia [Title/Abstract]) OR (Dystonia [Title/Abstract]) OR (Essential Tremor [Title/Abstract]) OR (Huntington’s Disease [Title/Abstract]) OR (Multiple System Atrophy [Title/Abstract]) OR (MSA [Title/Abstract]) OR (Myoclonus [Title/Abstract]) OR (Progressive Supranuclear Palsy [Title/Abstract]) OR (PSP [Title/Abstract]) OR (Unilateral Cerebral Palsy [Title/Abstract]) OR (UCP [Title/Abstract]) OR (Rett Syndrome [Title/Abstract]) OR (Secondary Parkinsonism [Title/Abstract]) OR (Spasticity [Title/Abstract]) OR (Tardive Dyskinesia [Title/Abstract]) OR (TD [Title/Abstract]) OR (Tourette Syndrome [Title/Abstract]) OR (Tics [Title/Abstract]) OR (Wilson’s Disease [Title/Abstract]) OR (Chorea [Title/Abstract]) OR (Epilepsy [Title/Abstract]) OR Seizure [Title/Abstract]))
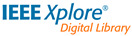	(((“Document Title”: Smartwatch) OR (“Document Title”: Smartwatches) OR (“Document Title”: Wristband) OR (“Document Title”: Wristbands) OR (“Document Title”: Bracelet) OR (“Document Title”: Bracelets) OR (“Document Title”: “Smart watch”) OR (“Document Title”: Wrist-worn) OR (“Document Title”: “Wrist device”) OR (“Document Title”: “Wrist devices”) OR (“Document Title”: Actigraph) OR (“Document Title”: “Apple watch”) OR (“Document Title”: Garmin) OR (“Document Title”: Fitbit)) AND ((“Document Title”: “Movement disorder”) OR (“Document Title”: “Gait disorder”) OR (“Document Title”: “Gait disorders”) OR (“Document Title”: “Gait”) OR (“Document Title”: “Parkinson’s disease”) OR (“Document Title”: Parkinson) OR (“Document Title”: Parkinson’s) OR (“Document Title”: “Movement disease”) OR (“Document Title”: Ataxia) OR (“Document Title”: Dystonia) OR (“Document Title”: “Essential Tremor”) OR (“Document Title”: “Huntington’s Disease”) OR (“Document Title”: “Multiple System Atrophy”) OR (“Document Title”: MSA) OR (“Document Title”: Myoclonus) OR (“Document Title”: “Progressive Supranuclear Palsy”) OR (“Document Title”: PSP) OR (“Document Title”: “Unilateral Cerebral Palsy”) OR (“Document Title”: UCP) OR (“Document Title”: “Rett Syndrome”) OR (“Document Title”: “Secondary Parkinsonism”) OR (“Document Title”: Spasticity) OR (“Document Title”: “Tardive Dyskinesia”) OR (“Document Title”: TD) OR (“Document Title”: “Tourette Syndrome”) OR (“Document Title”: “Wilson’s Disease”) OR (“Document Title”: Chorea) OR (“Document Title”: Epilepsy) OR (“Document Title”: Seizure))) OR (((“Abstract”: Smartwatch) OR (“Abstract”: Smartwatches) OR (“Abstract”: Wristband) OR (“Abstract”: Wristbands) OR (“Abstract”: Bracelet) OR (“Abstract”: Bracelets) OR (“Abstract”: “Smart watch”) OR (“Abstract”: Wrist-worn) OR (“Abstract”: “Wrist device”) OR (“Abstract”: “Wrist devices”) OR (“Abstract”: Actigraph) OR (“Abstract”: “Apple watch”) OR (“Abstract”: Garmin) OR (“Abstract”: Fitbit)) AND ((“Abstract”: “movement disorder”) OR (“Abstract”: “gait disorder”) OR (“Abstract”: “gait disorders”) OR (“Abstract”: “gait”) OR (“Abstract”: “parkinson’s disease”) OR (“Abstract”: Parkinson) OR (“Abstract”: Parkinson’s) OR (“Abstract”: “movement disease”) OR (“Abstract”: Ataxia) OR (“Abstract”: Dystonia) OR (“Abstract”: “Essential Tremor”) OR (“Abstract”: “Huntington’s Disease”) OR (“Abstract”: “Multiple System Atrophy”) OR (“Abstract”: MSA) OR (“Abstract”: Myoclonus) OR (“Abstract”: “Progressive Supranuclear Palsy”) OR (“Abstract”: PSP) OR (“Abstract”: “Unilateral Cerebral Palsy”) OR (“Abstract”: UCP) OR (“Abstract”: “Rett Syndrome”) OR (“Abstract”: “Secondary Parkinsonism”) OR (“Abstract”: Spasticity) OR (“Abstract”: “Tardive Dyskinesia”) OR (“Abstract”: TD) OR (“Abstract”: “Tourette Syndrome”) OR (“Abstract”: “Wilson’s Disease”) OR (“Abstract”: Chorea) OR (“Abstract”: Epilepsy) OR (“Abstract”: Seizure)))

**Table 2:** Some types of smartwatches are on the market.

	Brand	Model	Operating System	Main Sensors	Price
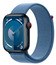	Apple	Series 9	watchOS	Electric heart rate ECG Third-generation optical heart rate Temperature Compass Altimeter High-g accelerometer High dynamic range gyroscope Ambient light	€ 439
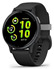	Garmin	VivoActive 5	GarminOS	Compass Accelerometer Thermometer Glonass GPS Pulse Ox Blood Oxygen Saturation Optical heartbeat Ambient light	€ 249
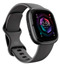	Google	Fitbit Sense 2	Fitbit OS	Glonass Optical heartbeat Altimeter Accelerometer GPS Ambient light Skin conductance	€ 219
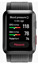	Huawei	Watch D	HarmonyOS 2.1	Accelerometer Gyroscope Optical heart rate Ambient light Skin temperature Differential pressure	€ 299
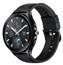	Xiaomi	Watch 2 Pro	Wear OS	Optical heart rate Accelerometer Gyroscope Ambient light Electronic compass Barometer Bioelectrical impedance	€ 211
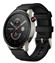	AmazFit	GTR 4	Zepp OS	Optical heart rate Accelerometer Gyroscope Blood Oxygen Saturation GPS	€ 199

**Table 3:** Some types of smart bands are on the market.

	Brand	Model	Dimensions	Main Sensors	Price
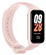	Xiaomi	Smart Band 8	48 mm × 22.5 mm × 10.9 mm	High precision 6-axis PPG heart rate Ambient light	€ 34
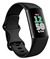	Google	Fitbit Charge 6	36.7 mm × 23 mm × 11.2 mm	3-axis accelerometer NFC Optical heartbeat Sp02 monitoring Ambient light	€ 129
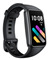	Honor	Band 7	43 mm × 25.4 mm × 10.9 mm	Optical heartbeat Sp02 monitoring NFC	€ 59
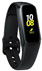	Samsung	Galaxy Fit	44.6 mm × 18.6 mm × 11.2 mm	3-axis accelerometer Gyroscope Optical heartbeat	€ 119
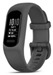	Garmin	VivoSmart 5	25.5 mm × 19.5 mm × 10.7 mm	3-axis accelerometer Optical heartbeat Sp02 monitoring Ambient light	€ 149

**Table 4:** Overview of the articles related to PD that investigated devices’ feasibility, usefulness, and acceptability.

Ref.	Commercial Devices	Kind of Smart Wrist Device	#End-Users	Data Availability
[28]	yes	Mobvoi, Ticwatch Pro	30	yes
[29]	yes	Apple Watch Series 2	51	subject to third-party restrictions
[30]	no		24	no
[31]	yes	Microsoft Band	75	no
[32]	yes	Pebble	953	yes
[33]	yes	Garmin Vivosmart 4	65	yes

**Table 5:** Overview of the articles related to PD symptom detection through smart wrist devices.

Ref.	Commercial Devices	Kind of Smart Wrist Device	#End-Users	Data Availability
[34]	yes	Empatica E4	25	no
[35]	no		40	no
[36]	yes	Android Smartwatch	13	yes
[37]	no		10	no
[38]	yes	Axivity AX-3	380	yes
[39]	yes	Axivity AX-6	8	no
[40]	yes	ActiGraph GT3X	60	no
[41]	yes	Microsoft Band 2	30	yes
[42]	yes	Axivity AX-3	12	no
[43]	no		83	no
[44]	yes	Apple Watch Series 2	13	subject to third-party restrictions
[45]	no		11	yes

**Table 6:** Overview of the articles related to PD assessment using smart wrist devices.

Ref.	Commercial Devices	Kind of Smart Wrist Device	#End-Users	Data Availability
[46]	yes	PD-watch	20	no
[47]	yes	PD-watch	12	no
[48]	yes	Verily Study Watch	96	yes
[49]	yes	Huawei Watch 2	40	yes
[50]	yes	Android Smartwatch	18	yes
[51]	no		154	yes
[52]	yes	KinetiSense	15	no
[53]	yes	Mobvoi TicWatch E	16	yes
[54,55]	yes	Clario Opal, Apple Watch 4 and 5	82	yes
[56]	no		100	no

**Table 7:** Overview of the articles related to PD progress monitoring through smart wrist devices (N.A. stands for Not Available).

Ref.	Commercial Devices	Kind of Smart Wrist Device	#End-Users	Data Availability
[57]	no		N.A.	no
[58]	no		N.A.	yes
[59]	no		10	no
[60]	yes	Apple Watch Series 4	21	yes
[61]	yes	Mobvoi, Ticwatch Pro	28	yes
[62]	yes	Kronowise 3.0	24	no
[63]	yes	Gaitup Physilog 4	20	yes
[64]	yes	Motorola G 360	316	yes
[65]	yes	Apple Watch	343	subject to third-party restrictions
[66]	yes	TED bracelet	52	no
[67]	yes	Axivity AX-3	34	no

**Table 8:** Overview of the articles related to PD and smart wrist devices, not categorized in previous subsections.

Ref.	Commercial Devices	Kind of Smart Wrist Device	#End-Users	Data Availability
[68]	no		N.A.	yes
[69]	yes	Apple Watch Series 4	318	subject to third-party restrictions
[70]	yes	Microsoft Band	200	no
[71]	no		10	no
[72]	yes	LG-W100	15	yes
[73]	no		18	no
[74]	yes	Apple Watch	68	subject to third-party restrictions
[75]	no		N.A.	no
[76]	yes	Verily Study Watch	149	yes
[77]	yes	Clario Opal, Apple Watch 4 and 5	40	yes
[78]	yes	GENEActiv	30	no
[79]	no		21	no
[80]	no		191	no
[81]	yes	Apple Watch Series 3 and 4	450	subject to third-party restrictions
[82]	yes	Verily Study Watch	388	yes
[83]	yes	Parkinson’s KinetiGraph	166	no
[84]	yes	ActiGraph GT9X Link	N.A.	no
[85]	no		N.A.	no
[86]	yes	Apple Watch Series 4	318	subject to third-party restrictions
[87]	yes	Garmin Vivosmart4	2	yes
[88]	yes	Opal APDM	80	no
[89]	yes	Opal APDM	154	no
[90]	yes	Verily Study Watch	370	no
[91]	yes	Parkinson’s KinetiGraph	61	yes
[92]	yes	Smartwatch3 (SW3) Sony	22	no
[93]	no		N.A.	yes
[94]	yes	Fitbit Sense and Empatica E4	17	no
[95]	yes	Mbientlab MetaWear, Apple Watch 2, Huawei watch	10	yes
[96]	yes	Pebble	1000	yes

**Table 9:** Overview of the articles that investigated epilepsy and seizure detection through Empatica smart wrist devices.

Ref.	Commercial Devices	Kind of Smart Wrist Device	#End-Users	Data Availability
[97]	yes	Empatica E4	14	yes
[98]	yes	Empatica E4	243	no
[99]	yes	Empatica E4 and Embrace	9	no
[100]	yes	Empatica E3 and E4, iCalm	69	no
[101]	yes	Empatica E4	38	yes
[102]	yes	Empatica E4	11	no
[103]	yes	Empatica E4	30	no
[104]	yes	Empatica E4	6	no
[105]	yes	Empatica E4	174	no
[106]	yes	Empatica E4	11	yes
[107]	yes	Empatica E4	69	no
[108]	yes	Empatica E4	32	no

**Table 10:** Overview of the articles that investigated epilepsy and seizure detection through other smart wrist devices (commercial and non-commercial).

Ref.	Commercial Devices	Kind of Smart Wrist Device	#End-Users	Data Availability
[109]	yes	Apple Watch Series 3, 4 and 5	106	subject to third-party restrictions
[110]	yes	Fitbit	13	yes
[111]	yes	Fitbit Charge 2	40	no
[112]	yes	Apple iPod touch	79	subject to third-party restrictions
[113]	yes	FitBit Charge 3, 4 and 5	12	yes
[114]	no		N.A.	no
[115]	yes	N.A.	79	no
[116]	yes	Apple Watch	999	subject to third-party restrictions
[117]	yes	SmartMonitor Smartwatch	41	no
[118]	yes	SmartMonitor Smartwatch	10	no
[119]	yes	Nightwatch	18	no
[120]	yes	Fitbit	11	yes
[121]	yes	Apple Ipod touch	11	subject to third-party restrictions
[122]	yes	Nightwatch	34	yes
[123]	yes	Epi-Care Free	1	no
[124]	yes	N.A.	1	no
[125]	yes	Garmin	316	no
[126]	yes	Shimmer3	11	no

**Table 11:** Overview of the articles that investigated essential tremor through smart wrist devices.

Ref.	Commercial Devices	Kind of Smart Wrist Device	#End-Users	Data Availability
[127]	yes	Pebble	8	no
[128]	no		41	no
[129]	yes	Cala Trio	276	no
[130]	yes	Cala One	77	no
[131]	yes	Pebble	20	no
[132]	yes	GENEActiv© Original	41	yes
[133]	no		4	no
[134]	yes	N.A.	219	no
[135]	yes	Shimmer3	33	yes
[136]	no		205	no
[137]	yes	Sony Smartwatch3	34	no
[138]	yes	Cala Trio	321	no
[139]	no		12	no

**Table 12:** Overview of the articles that investigated CP/UCP through smart wrist devices.

Ref.	Commercial Devices	Kind of Smart Wrist Device	#End-Users	Data Availability
[140]	yes	wGT3X-BT Actigraph and Mbientlab MetaMotion	10	no
[141]	yes	iWown i5 Plus	14	yes
[142]	yes	N.A.	18	no
[143]	yes	N.A.	11	no
[144]	yes	Axivity AX3	6	no
[145]	yes	Actigraphy GT9X Link	25	yes
[146]	yes	Actigraphy GT1M	11	no
[147]	yes	ActiGraph GT9X-BT	29	yes
[148]	yes	ActiGraph GT3X+	101	no

**Table 13:** Overview of the articles that investigated other movement disorders through smart wrist devices.

Ref.	Commercial Devices	Kind of Smart Wrist Device	#End-Users	Data Availability
[149]	yes	N.A.	15	no
[150]	yes	Motorola Moto G 360	160	yes
[151]	yes	Activinsights GENEActiv	64	yes
[152]	yes	Shimmer3	20	yes
[153]	no		18	yes
[154]	yes	Mobvoi TicWatch E	18	no
[155]	yes	Caretronic wristband	17	yes
[156]	yes	GENEActiv Original	31	yes
[157]	No		135	no
